# Decoding the microbiome: artificial intelligence-targeted gut microenvironment breakthroughs in personalized cancer therapy

**DOI:** 10.1080/19490976.2026.2672791

**Published:** 2026-05-29

**Authors:** Jingwen Liu, Pu Zhao, Deming Jiang, Shuyan Li, Chaoqiao Jin, Dingting Xu, Xiaoying Wang, Yan Chen, Bufu Tang, Xudong Qu

**Affiliations:** a Department of Gastroenterology, the Second Affiliated Hospital, Zhejiang University School of Medicine, Hangzhou, China; b Fourth Clinical College, China Medical University, Shenyang, China; c Laboratory Animal Center, Zhejiang University, Hangzhou, China; d Department of Interventional Radiology, Zhongshan Hospital, Shanghai Institute of Medical Imaging, National Clinical Research Center of Interventional Medicine, Fudan University, Shanghai, China

**Keywords:** Artificial intelligence, gastrointestinal microbiome, cancer, colorectal cancer (CRC), precision treatment, precision medicine

## Abstract

The gut microbiome functions as a key regulator of tumorigenesis and progression, thereby modulating tumor development and treatment outcomes (including chemoresistance, immunotherapy efficacy, and adverse effects) through its influence on the immune microenvironment and metabolite-mediated signaling pathways. Recent advances in multiomics technologies (metagenomics, metabolomics, and transcriptomics) have generated large-scale, comprehensive, and heterogeneous datasets whose complexity exceeds the capabilities of manual analysis, thus necessitating the implementation of artificial intelligence-based approaches. This review systematically examines the crucial role of the gut microbiome in tumorigenesis, with particular emphasis on colorectal cancer (CRC), specifically addressing its utility as a diagnostic and prognostic biomarker. Furthermore, building upon existing applications of artificial intelligence (AI) in microbiome research and cancer diagnosis and treatment, this review presents an AI-driven precision intervention framework and delineates personalized treatment strategies.

## Graphical abstract

**Figure:**
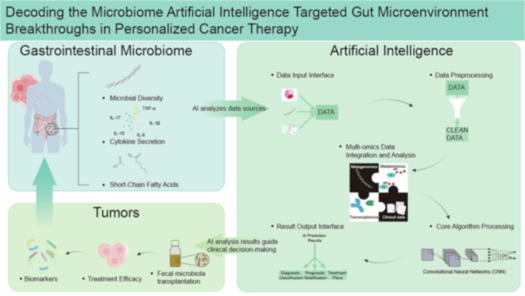

## 1. Introduction

The intricate interplay between gut microbiota and cancer development has garnered significant attention as a critical frontier in contemporary oncology research. The gut microbiota plays a pivotal role in regulating human health and influencing tumorigenesis, cancer progression, and therapeutic outcomes. Multiple mechanisms underlie gut microbiota's modulation of tumor development, including immunomodulation,^1^ ^,^ ^2^ metabolite-mediated signaling,^3^ ^,^ ^4^ and direct cellular stimulation of tumor cells,^5^ ^,^ ^6^ as emerging evidence demonstrates. These mechanisms highlight the dichotomous nature of gut microbiota in cancer biology, characterized by both protumorigenic activities and tumor-suppressive functions within specific tumor microenvironmental contexts.^7^ ^,^ ^8^

The advancement of precision medicine has underscored the critical importance of personalized approaches in cancer treatment.^9^ Precision medicine crafts personalized therapeutic interventions based on comprehensive patient profiling. This approach integrates genetic makeup, disease phenotypes, and lifestyle factors to optimize treatment outcomes while minimizing adverse reactions.^10^ Emerging research has established that gut microbiota substantially impacts cancer therapeutic responses, both in immunotherapy and chemotherapy, unveiling a promising pathway for tailored oncological interventions. For example, the machine learning model developed by De Rosa et al. based on gut microbial characteristics can predict patient responses to immune checkpoint inhibitor (ICI), enabling microbiome-based therapeutic selection.^11^ Furthermore, fecal microbiota transplantation (FMT) using microbiota extracted from ICI responders has demonstrated successful clinical application, restoring sensitivity to programmed death-1 (PD-1) inhibitors in patients with refractory melanoma and colorectal cancer, showcasing direct translational value.^12^ ^,^ ^13^ These advances indicate that gut microbiome analysis and modulation are being integrated into real-world precision oncology frameworks for risk stratification, drug response prediction, and personalized combination therapy design.

Therefore, therapeutic modulation of the gut microbiota for optimizing cancer treatment strategies has emerged as a crucial research direction in personalized medicine.^14^ The gut microbiota's intrinsic sophistication and variability, alongside its complex relationships with individual health and disease manifestations,^15^ result in massive and multidimensional datasets. Conventional analytical approaches demonstrate significant limitations in their ability to comprehensively analyze such complex data, thereby impeding the identification of underlying associations and patterns, and subsequently constraining the development of personalized, microbiota-targeted interventions for cancer therapy.

Current progress in artificial intelligence (AI) capabilities has delivered innovative analytical platforms and computational methods, enabling effective processing of these large-scale datasets. Advanced machine learning and deep learning algorithms enable researchers to identify microbial biomarkers correlated with tumorigenesis and treatment response from large-scale microbiome datasets.^16^ These computational approaches enhance patient care through two key mechanisms: anticipating treatment responses and enabling the advancement of novel therapeutic strategies. Specifically, a recently developed AI-integrated gut-on-a-chip system has demonstrated efficacy in screening functional probiotics for enteritis treatment.^17^ AI-driven methods enable the synthesis of multiomics data, unraveling the intricate relationships between the intestinal microbiome and immune response, which advances the implementation of precision medicine. By delving deeper into the complex link between the microbiome and host metabolism, researchers might unearth possible biological pathways and therapeutic targets, providing fresh perspectives on the tumor microenvironment.^18^

The dichotomous roles of the gut microbiota (protumorigenic and anti-tumorigenic) in cancer treatment and its therapeutic applications in personalized medicine present both unprecedented opportunities and significant challenges for oncology. Moreover, the implementation of AI technologies in analyzing gut microbiota-related data facilitates both a more comprehensive understanding of microbiota-cancer interactions and robust support for developing personalized cancer treatment strategies, thus advancing cancer therapy toward enhanced precision and efficacy.

Thus, this review addresses the following core question: How can artificial intelligence be leveraged to decode the complexity of the gut microbiome, and how can this knowledge bridge the gap between fundamental microbiome science and actionable, personalized cancer therapeutics?

We focus particularly on colorectal cancer as a paradigm, while also integrating evidence from other malignancies to provide a broader translational perspective. Through a critical synthesis of current advances and challenges, this review aims to chart a pathway toward AI-driven, microbiome-informed precision oncology.

## 2. Impact of the gut microbiota on tumors

### 2.1. Composition and function of the gut microbiota

The gut microbiota, collectively known as the intestinal microbiome, represents the human body's most sophisticated microbial community, harboring vast numbers of microorganisms that span bacteria, fungi, archaea, and viruses. These microorganisms constitute a highly diverse ecological community within the human gut, harboring a collective gene count that exceeds the human genome by more than 100-fold.^19^ ^,^ ^20^ This extraordinary microbial diversity not only reflects the intricate nature of the intestinal microbiome but also emphasizes its essential role in maintaining physiological balance.^21^ ^,^ ^22^ Under physiological conditions, the gut microbiota demonstrates high stability and adaptability, maintaining resilience against environmental perturbations and pathogenic invasion.^15^ Disruptions to this physiological equilibrium, particularly through dietary changes^23^ or exposure to antimicrobial agents,^24^ can trigger substantial shifts in the intestinal microbiome's composition and functionality, ultimately impairing host health.^25^ ^,^ ^26^

**Figure 1.**
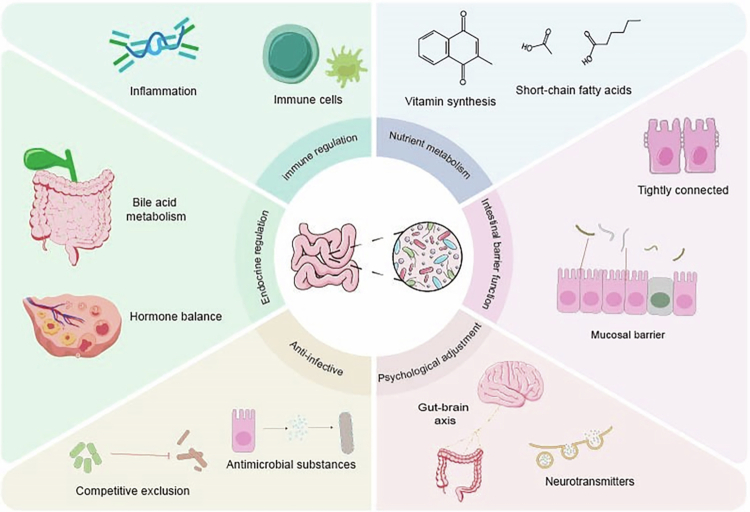
Gut microbiota orchestrate fundamental physiological processes in human health: the figure delineates the multifaceted contributions of intestinal microbiota to human physiological homeostasis. At the immunological level, the microbiome modulates immunological homeostasis through sophisticated immune regulation and anti-inflammatory mechanisms. In metabolic processes, the microbial community facilitates bile acid biotransformation and mediates SCFA biosynthesis, establishing crucial metabolic networks. The microbiota further maintain intestinal homeostasis via endogenous vitamin biosynthesis and regulation of epithelial barrier integrity. The microbiome additionally mediates bidirectional gut‒brain communication through neurotransmitter synthesis, influencing neuroendocrine regulation. Moreover, these microorganisms establish colonization resistance against pathogens through competitive exclusion and antimicrobial compound production. This intricate network of microbiota-mediated mechanisms collectively orchestrates systemic physiological homeostasis.

The gut microbiota has been extensively documented to play a fundamental role in maintaining human health (Figure 1), with particular emphasis on energy metabolism. Intestinal bacteria synthesize short-chain fatty acids (SCFAs) through the anaerobic fermentation of nondigestible dietary fiber. SCFAs exert dual regulatory functions: modulating host endocrine and immune signaling pathways via G protein-coupled receptors (notably G Protein-Coupled Receptor 41/43 (GPR41/43)) and mediating epigenetic modifications through modulation of histone acetyltransferases (HATs) and histone deacetylases (HDACs).^27^ Furthermore, the gut microbiota directly participates in host lipid metabolism, orchestrating lipid synthesis, uptake, and absorption, thus maintaining whole-body energy homeostasis.^28-30^

Functioning as the largest immune tissue in vertebrate organisms, the gastrointestinal tract sustains continuous interactions with an extensive microbial population—the gut microbiota—playing a crucial role in immune system modulation.^31^ Through a 17-week randomized controlled trial, Stanford University School of Medicine researchers demonstrated that a fermented food diet enhanced gut microbiota diversity (*P* < 0.01) while simultaneously reducing inflammatory markers, particularly Interleukin-6 (IL-6) (decreased by 28%, *P* = 0.003) and Interleukin-10 (IL-10), thereby validating the diet's direct role in modulating immune responses through microbiota remodeling.^32^

The recognition and verification of the microbiota–gut–brain axis model has revealed the intestinal microbiome as a crucial intermediary facilitating two-way communication between the digestive system and central nervous system (CNS), driving extensive research into its fundamental mechanisms. Using rodent models subjected to both dietary restriction and foot-shock stress paradigms, experimental evidence has shown a clear relationship between alterations in gut microbiota and binge-eating behavior. Through FMT experiments, Fan et al. demonstrated that dietary restriction and stress significantly modulate gut microbiota composition, specifically depleting beneficial bacterial populations, thereby inducing binge-eating behavior.^33^ Mechanistic studies have elucidated how the gut microbiota modulates feeding behavior via vagal signaling and specific neural circuits (such as the portal vein thrombosis (PVT)).

### 2.2. Clinical association between the gut microbiota and tumors

**Figure 2.**
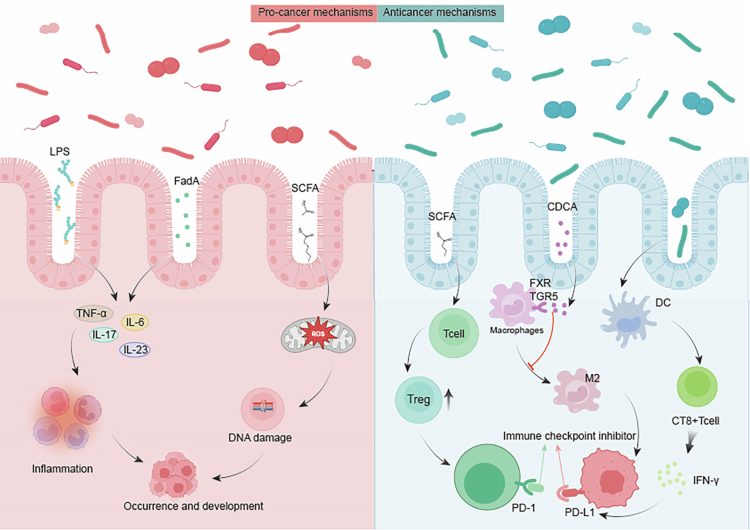
Procancer and anti-cancer mechanisms of gut microbiota in tumorigenesis: left panel (protumorigenic mechanisms): perturbation of microbiome homeostasis facilitates pathogenic bacterial expansion, leading to secretion of proinflammatory mediators including lipopolysaccharide (LPS), Fusobacterium adhesion A (FadA) adhesin, and oncogenic factors. These molecular triggers induce persistent inflammatory responses and cumulative DNA damage, synergistically promoting tumor progression. Right panel (antitumorigenic mechanisms): beneficial gut microbes produce SCFAs that activate GPR43/GPR109A receptors on T cells, promoting regulatory Treg differentiation for immune homeostasis while simultaneously upregulating PD-1 expression on tumor-infiltrating lymphocytes—a mechanism that synergizes with anti-PD-1 therapy to reverse T-cell exhaustion. Concurrently, secondary bile acids such as chenodeoxycholic acid (CDCA) signal through Farnesoid X receptor (FXR) and Takeda G protein-coupled receptor 5 (TGR5) receptors to inhibit M2 macrophage polarization of tumor-associated macrophages, thereby alleviating immunosuppressive microenvironments. Moreover, microbiota stimulate dendritic cell maturation, enhancing CD8⁺ T-cell infiltration into tumors and boosting Interferon-*γ* (IFN-*γ*) production; this cytokine in turn induces programmed ligand-1 (PD-L1) expression on tumor cells, establishing an immune checkpoint axis that can be effectively blocked by anti-PD-1/PD-L1 antibodies, ultimately strengthening antitumor immune surveillance and significantly improving response rates to immunotherapy.

The interaction between intestinal microbiome and cancer progression reveals a complex duality: it encompasses both tumor-promoting and tumor-suppressing pathways, reflecting the microbiota's multifaceted influence on metabolism, immune responses, and neurological activities (Figure 2). In terms of protumorigenic mechanisms, gut microbiota dysbiosis induces chronic inflammation, characterized by the release of proinflammatory cytokines,^32^ which facilitate tumor cell proliferation and survival. Additionally, specific gut bacterial species potentiate tumorigenesis and progression through the modulation of immunosuppressive pathways. Perturbations in metabolic homeostasis represent a critical mechanism through which the gut microbiota contributes to carcinogenesis. Intestinal microorganisms mediate the metabolic activation of triclosan(TCS) in the colonic environment, subsequently exacerbating colitis and colitis-associated colorectal cancer (CRC).^34^ Moreover, gut microbiota dysbiosis induces host metabolic perturbations, including obesity and insulin resistance,^35^ thereby establishing a permissive microenvironment conducive to tumorigenesis and disease progression.^36^

While the gut microbiota demonstrates protumorigenic potential, it simultaneously exhibits significant antineoplastic properties. Specifically, distinct microbial species demonstrate antitumor activity through the activation of host immune responses. Studies demonstrate how the intestinal microbiome defends against colitis and CRC through a cascade of events: the induction of IL-6 and IL-1β generation, which subsequently promotes T helper 17 cell proliferation.^37^ It is noteworthy that the role of Th17 cells in colorectal cancer is context-dependent: they can promote tumor progression through proinflammatory and proangiogenic functions while also participating in antitumor immunity under specific conditions.^38^ Moreover, the gut microbiota modulates antitumor drug efficacy through the regulation of host metabolic pathways. Notably, specific bacterial species facilitate the metabolism of ICIs, thereby potentiating their therapeutic efficacy.^39^ This microbiota-mediated drug metabolism mechanism presents novel opportunities for personalized medicine, specifically in optimizing antitumor therapeutic strategies through targeted microbiota modulation.

#### 2.2.1. Biomarkers

Changes in both the composition and function of the intestinal microbiome show significant correlations with cancer development and its subsequent progression.^40^ Through the secretion of metabolic mediators, immune modulation, and epigenetic modifications, the gut microbiota orchestrates tumor microenvironment remodeling, establishing its potential as a tumor biomarker^41^ ^,^ ^42^ (Table 1). In the context of CRC, *Fusobacterium nucleatum(Fn)* has emerged as a particularly significant clinical biomarker. Multicenter cohort analyzes have demonstrated that elevated *Fn* DNA abundance in CRC tumor tissues correlates with reduced overall survival, maintaining significance independent of tumor stage, microsatellite instability (MSI) status, and therapeutic intervention, thus validating its utility as an independent prognostic biomarker.^43^ ^,^ ^44^

**Table 1.** Cancer-associated gut bacteria: mechanisms and therapeutic potential.

Sites	Cancer-associated bacteria	Mechanisms	Potential for therapy
Esophagus	*Fusobacterium nucleatum* ^45^	Proinflammatory cytokine release suppressing anti-tumor immune responses.	High (Preclinical evidence in GI cancers; targeting Fn adhesion/invasion may enhance immunotherapy)
	*Porphyromonas gingivalis* ^46^	Activation of the TGFβ-SMAD/YAP/TAZ signaling pathway, providing a suitable tumor microenvironment.	Medium (Associative evidence; therapeutic relevance not yet demonstrated in vivo)
Stomach	*Dialister pneumosintes* ^47^	Altered metabolite production modifying the gastric mucosal environment.	Low (Mechanism unclear; limited therapeutic evidence)
	*Helicobacter pylori* ^48^ ^,^ ^49^	Group 1 Carcinogen: CagA protein induces epithelial damage, triggering gastric inflammation.	High (Established therapeutic target; eradication therapy reduces cancer risk)
	*Parvimonas micra* ^50^	Promotion of matrix metalloproteinase (MMP) secretion, accelerating tumor invasion.	Low (Co-occurs with oncogenic bacteria; direct therapeutic evidence lacking)
	*Peptostreptococcus stomatis* ^50^	Activation of the IL-6/STAT3 pathway, inhibiting apoptosis.	Medium (Associated with IL-6/STAT3 activation; bacterial targeting not yet validated)
	*Slackia exigua* ^51^	Bile acid metabolites promote cell proliferation.	Low (Limited evidence for direct therapeutic targeting)
	*Streptococcus anginosus* ^50^ ^,^ ^52^	Reactive oxygen species (ROS) production inducing gene mutations.	Low (ROS production implicated; no specific anti-bacterial strategy developed)
Colon and Rectum	*Bacteroides fragilis* ^53^	Secretion of B. fragilis toxin (BFT) disrupting the intestinal barrier and activating the NF-κB inflammatory pathway.	High (Targeting BFT toxin or NF-κB pathway suppresses tumorigenesis in preclinical models)
	*Escherichia coli* ^54^ ^55^	Production of colibactin toxin inducing DNA double-strand breaks, affecting DNA repair, promoting tumor cell proliferation.	High (Colibactin inhibitors under development; potential for chemoprevention)
	*Enterococcus faecalis* ^56^	Superoxide production leading to chromosomal instability.	Medium (Associated with oxidative DNA damage; specific targeting not established)
	*Fusobacterium nucleatum* ^57^ ^,^ ^58^	Key bacteria: Disruption of the intestinal epithelial barrier, damage to cellular DNA, activation of oncogenic signaling pathways, promotion of tumor cell metastasis, and promotion of inflammatory responses.	High (Multiple therapeutic strategies: antibiotics, immunotherapy, Fn-targeted vaccines)
	*Peptostreptococcus stomatis* ^59^	Synergistic promotion of tumor growth with *Fn*.	Low (Frequently co-occurs with Fn; therapeutic value uncertain)
Pancreas	*Acinetobacter* ^60^	Activation of the TLR4 pathway promoting fibrosis.	Low (Limited therapeutic evidence; mainly as a biomarker)
	*Helicobacter pylori* ^61^ ^,^ ^62^	Induction of chronic pancreatitis → pancreatic intraepithelial neoplasia.	Low (Epidemiological association inconsistent; therapeutic relevance unproven)
	*Pseudomonadales* ^63^	Promotion of immunosuppressive microenvironment (increased Treg cells).	Low (Taxonomic resolution too broad; functional role unclear)
Gallbladder	*Helicobacter pylori* ^64^	Gallstone formation → chronic cholecystitis → carcinogenesis.	Medium (Plausible biological link; clinical benefit not yet demonstrated)
	*Salmonella Typhi* ^65^ ^,^ ^66^	Risk in chronic carriers: Abnormal bile acid metabolism → gallbladder epithelial hyperplasia.	Medium (Antibiotic eradication may reduce cancer risk in carriers)
Bile Ducts	*Helicobacter spp* ^67^	Induction of bile duct epithelial DNA damage and high IL-8 expression.	Medium (Detected in bile; causal role and druggability remain uncertain)
	*Klebsiella pneumoniae* ^68^	Production of *β*-glucuronidase leading to secondary bile acid accumulation.	Medium (Targeting *β*-glucuronidase or bile acid metabolism may be therapeutic)
	*Ruminococcus Gnavus group* ^68^	Expression of B-cell superantigens, leading to excessive IgA production and exacerbating inflammatory responses.	Low (Mechanism unclear; therapeutic potential under investigation)

Multicohort metabolomic analyzes revealed distinctive alterations in eight gut microbiota–serum metabolome (GMSM) panel metabolites associated with CRC and adenoma development. Integration of targeted and untargeted metabolomics approaches demonstrated that this metabolite panel effectively discriminates between CRC, adenomas, and normal samples, achieving an area under the curve (AUC) of 0.93 with sensitivity and specificity exceeding 85%.^69^ Regarding CRC early detection, gut microbiota markers have demonstrated significant diagnostic potential. Metagenomic sequencing analysis in a multicenter study identified 20 CRC-specific bacterial signatures, including B. fragilis and *P*. anaerobius. With an AUC of 0.89 for distinguishing CRC from healthy controls and 76% sensitivity in early adenoma detection, this integrated bacterial signature model demonstrated robust diagnostic performance in the validation cohort.^70^ Notably, gut fungal dysbiosis (such as increased abundance of Malassezia) has also been shown to be associated with CRC progression and resistance to PD-1 inhibitors. This dysbiosis modulates the tumor immune microenvironment through *β*-glucan-mediated M2 macrophage polarization.^71^ ^,^ ^72^

The anatomical location of CRC tumors serves as a crucial prognostic indicator, substantially impacting microbial community composition and biomarker performance. Two key bacterial patterns emerge along the CRC sites: *Firmicutes* showing increasing abundance from right to left colon, while *Bacteroidetes* exhibit the opposite gradient. This location-specific distribution enables more accurate diagnostic biomarkers (AUC = 91.38%) compared to location-independent approaches (AUC = 82.92%). Location-based microbiota signature models, exemplified by the location gut index, enable prediction of differential chemotherapeutic responses.^73^

The diagnostic utility of gut microbiota signatures extends beyond CRC. In lung cancer surveillance, alterations in gut microbial profiles, particularly perturbations in *Prevotella* and *Ruminococcus* abundance, serve as predictive indicators for early-stage disease.^74^ Research on intracranial aneurysm (IA) has revealed stage-specific alterations in gut microbiota taxonomy, functional characteristics, and associated metabolomic profiles. Notably, indoxyl sulfate (IS), a tryptophan-derived metabolite, demonstrates discriminatory potential between ruptured intracranial aneurysm (RIA) and unruptured intracranial aneurysm (UIA) phenotypes.^75^ Additionally, gut microbiota modulates the tumor immune microenvironment via bile acid-mediated signaling, facilitating hepatocellular carcinoma progression.^76^ ^,^ ^77^ Recent advances have emerged in characterizing gut microbiota-based clinical subtypes in breast cancer.^78^

#### 2.2.2. Tumor treatment efficacy

**Figure 3.**
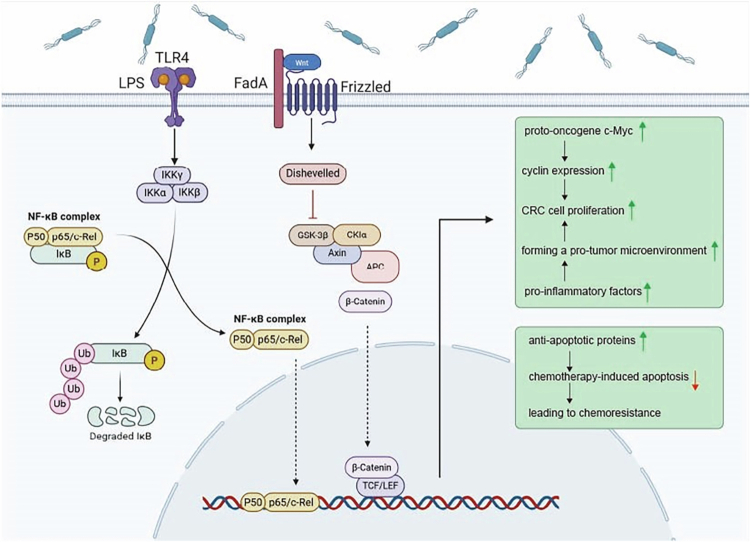
Mechanism by which *Fn* promotes CRC cell proliferation and chemotherapy resistance: *Fn* initiates molecular interactions with host cell E-cadherin through its adhesin FadA, destabilizing the E-cadherin/β-catenin complex integrity. This molecular disruption facilitates *β*-catenin nuclear translocation and activation, activating the canonical Wnt/β-catenin signaling cascade. Consequently, the transcriptional activation of downstream proto-oncogenes (c-Myc, Cyclin D1) drives enhanced CRC cell proliferation. Concurrently, *Fn*-derived LPS engages with TLR4 receptors, triggering NF-κB pathway activation. This signaling cascade induces the expression of proinflammatory cytokines (IL-6, tumor necrosis factor-alpha (TNF-*α*)) and antiapoptotic proteins, thereby attenuating chemotherapy-induced apoptotic responses and conferring chemotherapy resistance. The convergence of these molecular mechanisms significantly contributes to CRC progression and adverse clinical outcomes.

Beyond its strong links to tumor initiation and development, the gut microbiota significantly impacts how patients respond to cancer therapy.^63^ Research has demonstrated that the intestinal microbiome's composition plays a crucial role in determining both the effectiveness and adverse effects of anticancer medications.^79-82^ Orally administered cancer drugs reach the intestine. The abundant microorganisms in the intestine collectively encode genes 150 times that of the human genome, and their rich enzyme repertoire (such as nitroreductase and *β*-glucuronidase) can directly modify the molecular structure of chemotherapeutic drugs, leading to enhanced or inactivated drug activity.^83^ Specifically, *Fn* induces CRC cell proliferation and chemoresistance via toll-like receptor 4 (TLR4)/nuclear factor Kappa B (NF-κB) pathway activation, whereas its selective inhibition potentiates oxaliplatin efficacy^43^ ^,^ ^84^ (Figure 3). The microbiota-host metabolic interplay offers novel opportunities for precision oncology in CRC management. Metabolomic analyzes reveal suppressed butyrate metabolism and elevated secondary bile acids, particularly deoxycholic acid, in CRC patients. These metabolic changes diminish the effectiveness of PD-1/PD-L1 checkpoint inhibitors by altering both cytotoxic T-cell function and PD-L1 expression levels.^41^ ^,^ ^76^ Conversely, a microbiota profile rich in SCFAs (such as Faecalibacterium prausnitzii abundance >1.2%) can enhance tumor antigen presentation, increasing the objective response rate (ORR) of immunotherapy from 18% to 45%.^72^ ^,^ ^85^ The recently developed “gut ecology index” (GEI) further confirms that CRC patients in the high-GEI group (score ≥6.5) receiving PD-1 inhibitor combined with chemotherapy have a median progression-free survival (mPFS) of 14.2 months, significantly better than the low-GEI group (5.8 months, hazard ratio (HR) = 0.41).^72^ ^,^ ^85^ ^,^ ^86^

## 3. Applications of AI in the gut microbiota

### 3.1. Progress in core AI technologies

AI technologies are revolutionizing the scientific discovery process, facilitating hypothesis generation, experimental design optimization, high-dimensional data analysis, and enabling novel insights beyond conventional methodological approaches.^87^ ^,^ ^88^

#### 3.1.1. Progress in core technologies

Machine learning (ML) and deep learning (DL) constitute the foundational technologies of AI. Their capacity to explicitly address important issues in gut microbiota-tumor research is just as valuable as their strong computational capabilities. Supervised learning models (such as Support vector machines (SVM) and eXtreme gradient boosting (XGBoost)) utilize labeled data for training. They excel at processing high-dimensional, sparse metagenomic data by identifying optimal decision boundaries to classify gut microbiota data across different disease states, thereby providing a powerful tool for early diagnosis.^89-91^ However, its performance is highly dependent on the quality of annotated data, and when clinical samples are difficult to obtain, the model's generalization ability is limited.

Unsupervised learning algorithms identify intrinsic patterns in unlabeled data through cluster analysis, partitioning data points based on inherent similarities. Notably, K-means clustering stratifies patients into distinct subgroups based on microbiota compositional and functional similarities, facilitating the identification of disease-associated microbial signatures.^92^ However, interpreting the results of such methods often requires subsequent biological validation to avoid falling into data-driven illusions.

DL architectures emulate neural information processing through hierarchical network structures, demonstrating superior capability in extracting complex patterns from high-dimensional data.^93^ These architectures excel in processing diverse biomedical data modalities, including imaging, text analytics, and genomic sequences. Convolutional neural networks (CNNs) represent the predominant DL architecture for medical image analysis. The CNN architecture comprises hierarchical layers—input, convolutional, pooling, and fully connected layers—enabling automated feature extraction. In microbiota image analysis, convolutional layers perform feature extraction, pooling layers facilitate dimensionality reduction, and fully connected layers execute classification or regression tasks.^94^ ^,^ ^95^ Recurrent neural networks (RNNs) and their variants (long short-term memory (LSTM), gated recurrent unit (GRU)) specialize in sequential data processing, capturing temporal dynamics in microbiota evolution. LSTM and GRU architectures incorporate gating mechanisms to address the vanishing gradient challenge, enabling robust modeling of long-term dependencies in microbiota dynamics and treatment response prediction.^96^ Despite the powerful performance of DL models, their “black box” nature makes decision-making logic difficult to trace, posing a significant challenge in clinical scenarios requiring high interpretability (Table 2).

**Table 2.** Core technical principles and applications.

Technical categories	Core principles	Strengths	Limitations	Typical applications
Deep Learning (DL)	Utilizing deep neural networks to process and analyze large datasets, automatically identifying patterns and solving complex problems.^97^ ^,^ ^98^	Automatic feature extraction; well-suited for high-dimensional and complex data; strong end-to-end learning capability.	Requires large amounts of training data; High computational costs; “Black box” characteristics make decision logic difficult to trace.	Classification, functional annotation, and disease prediction of gut microbiota.^99^
Convolutional Neural Networks (CNNs)	Local connectivity and weight sharing extract spatial features, while pooling layers reduce dimensionality.^100^	Excellent at capturing spatial features; effective for image and structured data; weight sharing reduces overfitting.	Requires a large number of annotated images; sensitive to input dimensions; poor interpretability.	Colony morphology recognition and endoscopic image analysis.^94^ ^,^ ^101^ ^,^ ^102^
Recurrent Neural Networks (RNN)	Processing sequential data, capable of capturing dynamic changes in time series.^103^	Ideal for sequential modeling; handles variable-length sequences; retains temporal memory of past inputs.	Gradient vanishing problem; slow training speed; difficulty in modeling long-term dependencies.	Simulation and prediction of dynamic changes in gut microbiota.^103^
Supervised Learning (XGBoost)	Gradient boosting framework combined with regularization and second-order Taylor expansion, optimizing the handling of high-dimensional sparse data.^104^	Highly effective with high-dimensional sparse data; incorporates regularization to prevent overfitting; fast training speed; relatively interpretable.	Limited feature extraction capability; sensitive to changes in data distribution.	CRC diagnosis and detection, prediction of characteristic microorganisms.^105-107^
Reinforcement Learning (RL)	Based on Markov Decision Processes, learning optimal strategies through the interaction of an agent with its environment to maximize cumulative reward.^108^ ^,^ ^109^	Suitable for dynamic decision-making problems; adapts to environmental changes; optimizes for long-term cumulative rewards.	Training instability; challenging reward function design; requires extensive trial and error.	Prediction of degradation and biosynthesis pathways of phenolic compounds in the gut microbiome.^110^ ^,^ ^111^
Federated Learning (FL)	Multiple participants collaboratively train a global model without sharing raw data through encrypted parameter exchange or model updates.^112^	Preserves data privacy; enables distributed model training; well-suited for multicenter collaborative studies.	High communication costs; Difficult to process heterogeneous data.	Integration of multiomics data in gut microbiota research, avoiding the export of raw data and protecting privacy.^113^
Transfer Learning (TL)	Finding and leveraging similarities between source and target domains.	Effective in low-data regimes (few-shot learning); accelerates model convergence; enhances cross-domain generalization.	Performance is poor when the source domain and target domain differ significantly.	Overcoming geographical limitations to achieve high-precision diagnosis of cross-regional tumor microbial markers.^114^
Generative Adversarial Networks (GANs)	Generating high-quality data through the adversarial and iterative optimization of two neural networks.	Capable of generating realistic synthetic data; increases dataset diversity; useful for data augmentation and simulation.	Training difficulty is high; potential for model collapse.	Simulation of the abundance distribution of microbial communities.^115^

It is worth noting that a single AI model often struggles to address the complexity of microbiota-host interactions. Therefore, ensemble learning approaches—such as random forests—effectively enhance the robustness of disease risk or treatment outcome predictions by integrating the outputs of multiple weak models. More importantly, the emergence of multimodal AI architectures enables the integration of heterogeneous data from metagenomics, metabolomics, transcriptomics, and even medical imaging. This facilitates the construction of more comprehensive “digital twin” models that closely approximate real biological systems—a critical pathway for uncovering the underlying mechanisms by which the microbiome influences tumorigenesis and progression.^116^ ^,^ ^117^

DL implementation in oncological research primarily follows two distinct methodological approaches. The first approach leverages graphical user interface-based platforms, such as QuPath and ilastik, which facilitate implementation without programming expertise. The second approach entails programmatic interaction with DL frameworks through scripting languages, primarily Python, enabling direct manipulation of architectures such as CNNs and transformers.^118^

#### 3.1.2. Emerging technological breakthroughs

Reinforcement learning (RL) constitutes a fundamental paradigm in intelligent decision-making and algorithmic optimization frameworks.^108^ ^,^ ^119^ Grounded in Markov decision process (MDP) theory, RL optimizes agent behavior through environment interaction to maximize expected cumulative rewards.^120^ Advanced RL approaches, namely Q-learning, the policy gradient, and the deep deterministic policy gradient, enable sophisticated optimization of microbiota matching strategies between donors and recipients.

Intelligent capsules integrate miniature pH sensors, temperature sensors, and gas detection modules. They transmit intestinal physiological parameters in real time via radio-frequency identification, combined with microfluidic chips to collect in situ microbial samples, enabling real-time assessment of microbiota metabolic activity and guiding personalized nutritional interventions.^121^ Meanwhile, nanosymbionts use pH-responsive hydrogels to encapsulate probiotics or chemotherapeutic drugs (such as irinotecan). These systems achieve targeted delivery to hypoxic tumor microenvironments (O₂ < 0.5%) via surface-functionalized ligands, including anti-EpCAM antibodies, enhancing therapeutic bioavailability and tumor penetration.^122^

### 3.2. Specific applications of AI

In AI-driven microbiome research, investigators typically adopt a systems biology approach rather than focusing on individual bacterial species or strains. The methodology centers on examining disease-associated perturbations in microbial communities and their metabolomic signatures. Researchers have implemented machine learning frameworks to infer absolute microbial load from relative abundance profiles. This approach has established microbial load as a critical determinant of microbiome variability, demonstrating both direct and indirect disease-mediated effects.^123^

#### 3.2.1. Data collection and processing

AI has emerged as a pivotal analytical framework for elucidating gut microbiota-CRC associations, advancing precision oncology through integrated data analytics and predictive modeling. Advanced biosensor-integrated capsule systems enable multiparametric monitoring of intestinal physiology (pH, temperature, pressure, gas composition) and microbiota sampling, generating comprehensive datasets for gastrointestinal disease diagnostics and surveillance.^121^ AI-powered automation platforms substantially enhance throughput and reproducibility in microbiome data acquisition. An integrated high-throughput culturomics platform, incorporating robotics and automated microscopy, enables parallel cultivation of >1000 intestinal strains per batch. Combined with CNNs for automatic recognition of colony morphology and growth kinetics, this increases strain isolation efficiency by 5 times, with a species identification accuracy of 98% (vs. 82% for traditional manual isolation).^91^ To address the batch effect problem in metagenomic data, a supervised nonnegative matrix factorization (sNMF) model decomposes the sample-species matrix to identify and correct technical variations introduced by experimental procedures (such as DNA extraction methods). This approach enhances inter-cohort correlation of Shannon diversity indices from 0.41 to 0.79.^96^ Machine learning analysis of these datasets has facilitated the identification of CRC-specific microbial signatures, including Bacteroides fragilis and Peptostreptococcus anaerobius. The integrated diagnostic model achieves an AUC of 0.91, with early adenoma detection sensitivity reaching 79%.^92^ ^,^ ^124^

However, despite improvements in data scale and quality, two critical methodological bottlenecks—data imbalance and small sample size constraints—continue to profoundly impact the final output of these technologies. First, at the sample level, although automated platforms have increased processing throughput, the construction of clinical cohorts remains frequently constrained by sample acquisition difficulties, resulting in relatively small total sample sizes (typically ranging from dozens to hundreds of cases). When confronted with the inherently “high-dimensional” nature of microbiome data (thousands of features), this small sample size readily triggers dimensionality catastrophe and overfitting, causing models to perform drastically worse in independent validation. Second, at the data distribution level, imbalance is prevalent: either disproportionate ratios between case and control groups (categorical imbalance) or critical rare microbial features (e.g., specific fungi or low-abundance communities) being obscured by abundant species (feature imbalance). For instance, even though the aforementioned comprehensive diagnostic model achieved an excellent AUC of 0.91 and identified CRC-specific microbial features like Bacteroides fragilis, we must critically evaluate its generalization capability: Is the model biased toward minority class samples (e.g., early adenoma patients)? Do its identified features overlook rare microbes that are functionally critical despite low abundance? Without addressing these questions, a high AUC value may merely reflect accurate identification of the majority class. The 79% sensitivity for minority classes (e.g., early adenomas) could still lack stability due to random fluctuations when absolute sample sizes are small.

Therefore, future data collection and processing workflows should not only pursue improvements in throughput and accuracy but also incorporate considerations for imbalanced and small-sample problems from the outset—such as balancing class distributions through oversampling techniques or introducing sensitivity analysis for rare features during feature selection. This approach provides a more robust data foundation for downstream machine learning modeling.

Despite automation enhancing data collection efficiency, reproducibility remains a critical challenge in microbiome bioinformatics analysis workflows. Reproducibility refers to whether consistent conclusions can be obtained from identical raw data using different analytical workflows. Current workflows exhibit significant variations across multiple stages: choice between operational taxonomic unit (OUT) and amplicon sequence variant (ASV) methods during feature table construction impacts species resolution; species annotation relies on different databases (e.g., Greengenes, SILVA), with consistency below 70%; selection of α/β diversity algorithms and differential abundance models (e.g., DESeq2, ANCOM-BC) alters final biomarkers. This heterogeneity directly leads to an abundance of “false positive” biomarkers and conflicting results across studies. The MBQC project revealed that 16 institutions using identical samples but different workflows produced significantly divergent community structures. To enhance reproducibility, the research community is promoting workflow standardization, containerization technology, open-source code and parameters, and recommending a multiworkflow consensus strategy—retaining only findings robust across multiple workflows.

#### 3.2.2. Health assessment and risk prediction

Machine learning approaches have demonstrated remarkable advances in microbiome-based disease assessment and risk stratification. Evidence from diverse studies demonstrates ML models' capacity to leverage microbiome signatures for individualized risk assessment and disease phenotyping across multiple conditions, including diabetes,^125^ ^,^ ^126^ Parkinson's disease,^127^ and inflammatory bowel disease (IBD).^128^

AI exhibits multifaceted utility in CRC research. Through integration of 2,320 fecal metagenomic profiles, AI-driven multiclassification models achieve robust discrimination of nine distinct disease phenotypes, including CRC, with an AUC of 0.89.^124^ Cross-cohort analyzes have revealed significant correlations between CRC-specific microbial signatures and perturbations in serum metabolomes. ML-based integration of multiomics data has achieved a 12% higher specificity in early CRC detection (AUC: 0.94), outperforming single-omics approaches (AUC: 0.82). The underlying mechanism encompasses SCFA deficiency-mediated NF-κB pathway activation and intestinal barrier dysfunction.^129^ Fungal communities, as integral components of the gut microbiome, play crucial roles in tumor immune microenvironment modulation. Aberrant *Candida* proliferation in lung cancer and CRC patients establishes a protumorigenic niche characterized by acidic pH and elevated lactate, promoting immunosuppression through IL-1β/Th17 axis activation.^130^ Fungal community-based random forest models demonstrate superior predictive capability for hepatic precancerous lesions compared to conventional clinical indicators (AUC: 0.82 vs. 0.65), validating fungal signatures as novel pancancer early warning biomarkers.^131^ Further investigations have revealed distinct CRC patient stratification into two subgroups based on gut microbial profiles. This stratification-based diagnostic model significantly enhances diagnostic precision, advancing personalized therapeutic strategies for CRC.^92^

In terms of CRC treatment, scientists have recently identified shared “beneficial microbiota characteristics” (such as enrichment of Bifidobacterium and Akkermansia) across cancer types (melanoma, nonsmall cell lung cancer, etc.). A newly developed microbial classification model predicts responses to PD-1/cytotoxic T-lymphocyte-associated protein 4 (CTLA-4) inhibitors, advancing patient stratification strategies in cancer immunotherapy.^132^ Analysis of transcriptomic data using machine learning predicts oxaliplatin adjuvant chemotherapy outcomes in stage II‒III colon cancer, revealing a substantial 23% improvement in 5-y disease-free survival among benefiting patients. The key molecular indicators reflect the functional state of DNA damage repair mechanisms.^133^

In the context of CRC prognostication, research has established a strong association between postchemotherapy bloodstream infection (BSI) risk and intestinal dysbiosis, characterized by diminished microbial diversity and aberrant expansion of opportunistic pathogens, particularly Enterococcus species. A machine learning model incorporating microbial diversity indices and Enterococcus abundance metrics demonstrates robust prediction of postchemotherapy BSI risk in CRC patients, achieving 90% sensitivity and specificity (AUC = 0.94),^134^ thus enabling timely therapeutic intervention.

*Fn*, for high-risk individuals with IBD-associated CRC, AI-driven endoscopy analysis systems (such as EndoBRAIN) have achieved objective quantification of mucosal inflammation. This system, by identifying pathological features with a Nancy index ≥ 2 (indicating high-grade dysplasia), demonstrates better diagnostic concordance (*κ* = 0.85) than traditional pathological assessment (*P* < 0.01).^135^ ^,^ ^136^ Combined with microbiome markers (such as *Fn* enrichment^137^ and copper-dependent cell death,^138^ this technology can establish a multimodal risk warning system and optimize CRC monitoring strategies (Table 3).

**Table 3.** Performance of AI models in microbiome-based cancer diagnostics.

Cancer types	Model applications:	Data types	Key findings	Clinical utility	Limitations
Multiple cancer types	Multiclass Model	Integrated data from 2320 fecal metagenomes^139^	Differentiation of 9 disease phenotypes, including CRC.	Enables multidisease differential diagnosis and early screening.	High model complexity; weak interpretability; requires large-scale validation.
CRC	Machine Learning integrating multiomics data	Metagenomic + serum metabolomic data^140^	Improved specificity in early CRC detection.	Enhances early CRC screening accuracy for high-risk populations.	Limited sample size; metabolic data stability affected by individual variation.
CRC	Random forest model	Gut microbial characteristics subgrouping patients^141^	Significantly improved diagnostic accuracy.	Improves diagnostic accuracy and pathological subtype identification.	Feature selection depends on training data; generalization requires external validation.
CRC	SHAP algorithm	Gut microbiome data^126^	Identification of specific bacteria significantly associated with CRC.	Provides interpretable biomarkers for mechanistic studies and target development.	Correlation does not imply causation; it requires experimental validation.
CRC	SVM Classifier	Metagenomic data	Improved model prediction accuracy and robustness through recursive feature elimination for gene selection.	Suitable for gene signature screening and high-dimensional data classification.	Sensitive to data distribution; high computational cost.
CRC (poor prognosis)	Machine learning model	Characteristics of gut microbiota dysbiosis (*Bacteroides/Enterococcus* ratio)	Prediction of bloodstream infection risk after chemotherapy.	Guides preventive interventions and personalized supportive care before chemotherapy.	Single-center cohort; requires prospective multicenter validation.
Early-stage HCC	Random forest model	Bacterial metagenomic data	Prediction of precancerous lesions in liver cancer.	Non-invasive early warning for high-risk populations.	Bacterial composition stability affected by diet/environment.
Lung cancer	Machine learning model	Specific bacterial markers (such as *Fusobacterium*)	Effective prediction of early lung cancer risk.	Microbiome-based biomarkers supplement imaging diagnostics for unclear cases.	Gut–lung axis mechanism unclear; confounded by smoking and environmental factors.
Recurrent ovarian cancer	Integrated analysis (including ML)	Tumor transcriptomic + fecal metabolomic + Gut microbiome data	Revealed the correlation between gut microbiome and metabolome changes and immunotherapy response.	Multiomics basis for immunotherapy response prediction and patient stratification.	Small sample size; multiomics integration methods not standardized; long clinical translation path.
Stage II–III colon cancer	Machine learning model	Gut microbiome + transcriptome^142^	Prediction of therapeutic benefit from oxaliplatin-based adjuvant chemotherapy.	Guides oxaliplatin-based adjuvant chemotherapy selection to maximize treatment benefit.	Microbiome not integrated (needs supplementation); requires prospective trials.

Although numerous studies have reported high accuracy rates for AI models in predicting tumor risk or treatment response, not all research has successfully replicated these findings. For instance, attempts to validate AI classifiers based on specific microbial biomarkers in independent cohorts revealed significantly diminished performance, highlighting the models' inadequate generalization capabilities.^143^ Furthermore, several clinical trials aimed at improving CRC patient outcomes through FMT or specific probiotic interventions have failed to achieve anticipated results. Research indicates that while FMT effectively alleviates diarrhea symptoms, its impact on long-term improvement in disease activity is limited, and some patients experience mild adverse reactions (e.g., bloating, nausea.^144^ The presence of these “negative” or “contradictory” findings precisely reveals the challenges facing current research: the complexity of the microbiome, substantial biological variability between individuals, and the nonspecific nature of interventions may all contribute to unpredictable outcomes.

#### 3.2.3. Multiomics data integration and analysis

**Figure 4.**
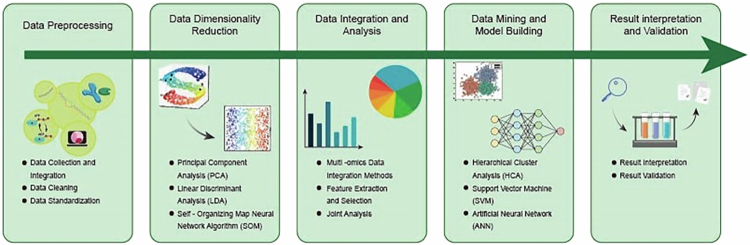
Fundamental steps for multiomics data integration and analysis: five main steps: data preprocessing, dimensionality reduction, data fusion and analysis, data mining and model construction, results interpretation and validation. In the data preprocessing stage, perform data collection and integration, data cleaning, and standardization. Next, perform dimensionality reduction through techniques such as principal component analysis (PCA), linear discriminant analysis (LDA), and the self-organizing feature mapping (SOM) neural network algorithm (SOM). Then, utilize multiomics data fusion methods, feature extraction and selection, and joint analysis to perform data fusion and analysis. In the data mining and model construction stage, techniques such as hierarchical clustering analysis (HCA), SVM, and artificial neural networks (ANNs) are used. Ultimately, perform interpretation and validation of results. This process helps extract meaningful information from complex data to support decision-making.

Furthermore, AI, by integrating endoscopic imaging, histopathology, and multiomics data,^145^ has opened up a multidimensional analytical paradigm for the precise diagnosis and treatment of CRC^146^ (Figure 4). A DL-based “Endoscopic-histomics” system (such as EndoHistoAI) can simultaneously analyze the microscopic structural characteristics and microbial metabolome profiles of the intestinal mucosa in CRC patients. Its AUC for differentiating early IBD-related CRC reaches 0.93, significantly better than single-modality diagnosis (*κ* consistency increased from 0.62 to 0.87). Research into IBD-to-CRC progression reveals that specific dysbiosis, particularly *Bacteroides spp* abundance exceeding 30%, promotes carcinogenesis by triggering epithelial-mesenchymal transition (EMT) through miR-215-5p suppression in intestinal epithelial cells.^147^ ^,^ ^148^ The metagenomic analysis tool SIAMCAT further confirms that CRC-specific biomarkers are continuously enriched in the adenoma-carcinoma sequence, while cross-disease shared markers (such as Bacteroides vulgatus) are associated with chemotherapy resistance (OR = 2.1, *P* < 0.01).^149^ ^,^ ^150^ By constructing a “microbiome–host” multiomics interaction network, AI models (such as IMOVNN) successfully locate key regulatory hubs in CRC—the synergistic activation of the quorum-sensing gene luxS and the host Wnt/β-catenin pathway. Its AUC for predicting tumor invasiveness is 0.88 (sensitivity 82%, specificity 91%).^148-150^ Moreover, AI-based dynamic modeling of metabolic networks enables quantification of how microbial metabolites regulate immune checkpoints in CRC. Secondary bile acids (deoxycholic acid > 60 μM) inhibit CD8+ T-cell function (reducing IFN-*γ* secretion by up to 70%) by activating the FXR receptor, while propionate (>100 μM) can reverse this effect and enhance the response rate to PD-1 inhibitors to 48% (vs. baseline 16%).^146^ ^,^ ^150^ These results underscore the essential role of integrated multiomics analysis in understanding CRC heterogeneity and enhancing precision treatment strategies. By collecting and integrating different omics data, researchers can uncover the intricate relationships between key players in the pathogenesis.

In recent years, various integrated AI frameworks have been successfully applied to multiomics data analysis, significantly enhancing predictive accuracy and biological interpretability. For instance, the OmniNet-Fusion model integrates multiomics data by combining CNNs, RNNs, and attention mechanisms to predict cancer drug responses, achieving 94.2% accuracy and an AUC-ROC of 0.96 on the validation set, demonstrating the immense potential of deep learning in precision drug therapy.^151^ Regarding interpretability, the MOGKAN framework integrates mRNA, miRNA, DNA methylation, and protein interaction network data based on Kolmogorov-Arnold network theory. It achieves 96.28% accuracy across 31 cancer classification tasks and validates its identified biomarkers through Gene Ontology and KEGG enrichment analysis.^152^

Despite the promising prospects of multiomics integration, its technical implementation poses significant challenges. First, dimensionality mismatch presents a primary obstacle: metagenomic data typically encompasses thousands of species features, while metabolomic data contains only hundreds of metabolites. This vast disparity in dimensionality necessitates specialized dimension reduction or embedding techniques (such as multiview autoencoders) to align the representational spaces of different data sources. Second, batch effects are pervasive. Data generated across different laboratories and sequencing platforms exhibit systematic biases. Without correction—such as employing sNMF—AI models are prone to learning technical noise rather than genuine biological signals, leading to failure in cross-cohort validation.^18^

More crucially lies the dilemma of causal inference. Most current AI models—including advanced deep learning approaches—remain fundamentally correlation-based tools. They can identify strong associations like “high Fusobacterium nucleatum abundance” and “chemotherapy resistance,” but cannot directly prove that *Fn* causes resistance. To overcome this limitation, researchers are integrating causal inference methods into AI frameworks. For instance, constructing causal networks linking microbes, metabolites, and host gene expression can reveal whether specific microbial communities influence host drug metabolism enzyme activity by regulating metabolic pathways (such as secondary bile acid synthesis), ultimately driving therapeutic outcomes.^153^ Although these methods are in their early stages, they represent an essential path from “correlation” to “causation” and are crucial for developing truly effective targeted microbiome therapies.

### 3.3. Functions of AI in diagnosis, prognosis, and treatment response prediction

When applying artificial intelligence to gut microbiome-oncology research, clearly defining the core predictive objectives of the model is crucial. Although diagnostic prediction, prognostic prediction, and treatment response prediction all rely on data modeling, they exhibit fundamental differences in clinical significance, temporal dimensions, modeling strategies, and validation approaches. Distinguishing these three categories helps avoid conceptual confusion and provides more precise positioning for the clinical translation of models.^154-156^

The purpose of diagnostic prediction models is to determine at a specific point in time whether a patient has a particular disease or condition based on their microbiome characteristics. These models essentially address a classification problem, with outputs typically representing “probability of disease” or “disease subtypes.” For instance, distinguishing colorectal cancer patients from healthy controls using fecal metagenomic data, or identifying gut microbial signatures associated with early-stage lung cancer.^53^ “Gold standard” validation for diagnostic predictions typically relies on comparison with established diagnostic methods such as pathological biopsy or imaging. The core challenge lies in distinguishing causality (whether microbiome changes are a cause or consequence of disease) and controlling for confounding factors (such as dietary or pharmaceutical interference with the microbiome).^157^ ^,^ ^158^

The purpose of prognostic prediction models is to forecast future disease progression outcomes for patients (such as recurrence risk, overall survival, disease-free survival) based on their current state, regardless of whether they receive specific treatments. These models reveal the natural history of the disease or the expected trajectory following standard therapy. Examples include predicting overall survival in colorectal cancer patients based on F. nucleatum abundance in tumor tissue, or forecasting bloodstream infection risk based on prechemotherapy gut dysbiosis characteristics.^43^ The core value of prognostic prediction models lies in risk stratification, aiding clinicians in identifying high-risk populations to develop more proactive monitoring or intervention strategies. Their validation requires long-term follow-up data and confirmation of stability across multicenter cohorts.

Therapeutic response prediction models (also known as predictive biomarker models) aim to forecast a patient's susceptibility or resistance to specific therapeutic interventions. These models serve as core tools in precision medicine, directly guiding treatment decisions and, for instance, predicting patient benefit from PD-1 inhibitor therapy based on gut microbial ecological topology indices, or identifying early-stage colon cancer patients responsive to oxaliplatin adjuvant chemotherapy using the colon oxaliplatin characteristics (COLOXIS) model.^159^ Validation of treatment response prediction must be grounded in randomized controlled trials or prospective cohorts involving therapeutic interventions. It is crucial to distinguish this from prognostic prediction—a robust treatment response prediction model should identify the interaction between “treatment-responsive” and “nonresponsive” populations.

Understanding the distinctions between these three is crucial for clinical practice: diagnostic models determine “whether treatment is needed,” prognostic models determine “the urgency and intensity of treatment,” while treatment response models determine “which treatment to choose.” It is noteworthy that an excellent prognostic biomarker may not necessarily be a good treatment response biomarker (e.g., high-risk prognostic patients may respond well to a certain therapy), and vice versa. Future AI models should evolve toward multifunctional capabilities, such as simultaneously outputting a patient's diagnostic classification, prognostic risk, and predicted response probabilities to multiple therapies through a multitask learning framework, thereby providing comprehensive support for clinical decision-making. This also requires researchers to clearly define their predictive objectives at the outset of model design and select appropriate modeling strategies and validation protocols accordingly.^44^

## 4. Personalized targeted modulation of the gut microbiota

### 4.1. Personalized treatment

As AI advances and our knowledge of intestinal microbiome-health interactions deepens, the evaluation and precise regulation of patients' microbial profiles and functions emerge as crucial components of future integrated, personalized healthcare approaches.^160^ ^,^ ^161^ A new machine learning model, developed by researchers, can forecast how food consumption affects blood glucose and triglyceride levels by analyzing individual parameters, such as the gut microbiome, helping to develop personalized dietary strategies^162^ and thus achieve personalized treatment. However, the first step in achieving personalized treatment is to improve the accuracy of diagnosis. AI technology can already accurately classify 22 known cancer types by analyzing somatic alterations^163^ and can also subclassify a single cancer (such as CRC).^16^ A pivotal role in tumorigenesis and disease progression is played by modifications in the intestinal microbiome and its metabolic compounds.^164^ By analyzing high-dimensional data of the gut microbiota, it is possible to successfully mine microbial markers closely related to tumorigenesis and progression. By integrating topological data analysis (TDA) and machine learning approaches based on intestinal microbiota systems, Derosa et al. identified a novel biomarker derived from gut microbiome ecological structure that accurately predicts immunotherapy outcomes and shows promise for directing personalized microbial interventions.^11^ DL approaches, particularly transformer models, have enabled the identification of potential diagnostic markers (MSI, BRAF, and KRAS)^165^ from metagenomic data. However, these discoveries reflect correlations rather than causation. Achieving precise personalized therapy requires detailed clarification of how gut microbiota mechanistically influences cancer.

However, the vast amount of gut microbiota data means that relying solely on the human brain for connection and integration is unrealistic. This is where the use of AI to integrate multiomics data plays an indispensable role. Multiomics data integration is the core of AI-driven personalized treatment.^113^ ^,^ ^148^ Through a systematic review of genome-wide association studies data from over 250,000 subjects (100,204 CRC cases and 154,587 controls), coupled with thorough genetic, transcriptomic, and methylation analysis of various tissues, including colonic mucosa, researchers identified 155 high-confidence genes associated with CRC risk, many previously unknown.^166^ In the field of immunotherapy, the Rosario team, after clinical trials, integrated tumor transcriptomic, fecal metabolomic, and gut microbiome data to reveal the impact of the tumor–immune–gut axis on the response to immunotherapy in patients with recurrent ovarian cancer. The researchers demonstrated that distinct alterations in both the intestinal microbiota and metabolic profiles correlate with immunotherapy outcomes, suggesting these factors may affect therapeutic effectiveness through regulation of the tumor immune environment.^167^

### 4.2. Microbiota modulation techniques

#### 4.2.1. Optimization of FMT

**Figure 5.**
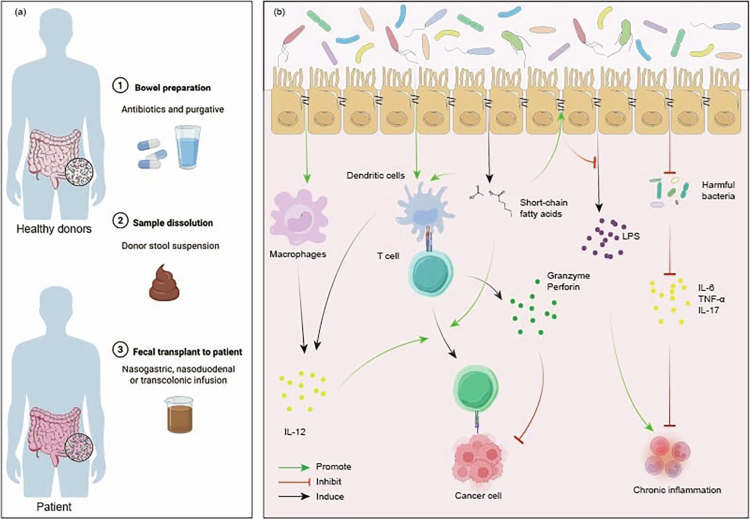
FMT basic procedure (a) and mechanism of action (b): panel a: illustrates the process of collecting fecal samples from healthy donors, processing the material, and transferring it into recipients to reestablish their gut microbiota. Panel b: details the pathways by which transplanted microorganisms produce beneficial outcomes: the intestinal microbiome facilitates immune cell maturation and activation through SCFAs and other metabolic products, while regulating immune responses through cytokines to enhance anti-tumor immunity.SCFAs interact with intestinal epithelial cells, strengthening mucosal barrier integrity. This prevents harmful substances from entering systemic circulation, thereby reducing systemic inflammation.

FMT, as an early treatment method, involves transplanting the entire gut ecosystem of a healthy donor into a patient, representing a significant avenue for altering the gut microbiota. This approach has been extensively investigated across multiple medical conditions and has demonstrated therapeutic efficacy in various types of cancer.^168-173^ (Figure 5) Research has demonstrated that transferring the intestinal microbiome from treatment responders to experimental models through FMT enhances the antitumor efficacy of PD-1 inhibition.^12^ ^,^ ^13^ ^,^ ^174^ Interestingly, F. nucleatum, typically known to drive CRC progression, can improve the response of microsatellite stable (MSS) CRC to PD-1 checkpoint inhibitors, despite these tumors usually being resistant to anti-PD-1 therapy.^175^ Experimental evidence indicates enhanced colonization efficiency of donor strains when corresponding species are pre-existing in the recipient microbiota.^176^ FMT outcomes are fundamentally dependent on recipient baseline microbiota composition, donor microbial community structure, and host immunological parameters. These findings underscore the necessity for individualized approaches throughout the FMT therapeutic pipeline.^177^

Although FMT has demonstrated efficacy in autoimmune diseases such as IBD, its clinical application still faces significant challenges. First, substantial individual differences between donors and recipients make the success rate and effectiveness of microbiota colonization difficult to predict. Second, as a therapy involving live biological agents, FMT's safety profile—including potential pathogen transmission risks—and long-term health impacts require evaluation through large-scale, prospective cohort studies with extended follow-up. Furthermore, the lack of standardized operating procedures—including preparation, storage, and administration methods for the bacterial suspension—remains a major bottleneck hindering its widespread adoption. Consequently, despite its theoretical potential to alter the host microbiome, FMT's clinical application in cancer treatment remains in the early exploratory phase, with a long journey ahead before it can become a routine therapeutic approach.

#### 4.2.2. Precision probiotic therapy

Probiotic supplementation represents an established approach for microbiota modulation, demonstrating significant implications in cancer therapeutics.^178-180^ AI enables analysis of gut microbial functional networks to identify probiotics with potential antineoplastic properties. Researchers have developed a microfluidic gut-on-chip platform for high-throughput screening of anti-inflammatory probiotics. The platform facilitates probiotic screening through three integrated steps: high-throughput coculture of intestinal epithelial cells with probiotics, automated sample analysis, and machine learning-based probiotic efficacy assessment. This streamlined approach circumvents the requirement for conventional in vitro and in vivo testing paradigms.^17^ Since chronic inflammation is considered a driver of CRC development,^135^ this technology is likely applicable to the treatment of IBD-associated CRC. Machine learning algorithms facilitate the design of optimized probiotic combinations with enhanced antineoplastic efficacy.^181^ ^,^ ^182^ Integration of in vitro gastrointestinal models with machine learning tools enables prediction of probiotic formulation metabolic profiles and therapeutic outcomes.^183^ AI-guided “nanosymbiotic” platforms, including pH-responsive microrobots, achieve precise probiotic delivery to hypoxic CRC regions (O₂ < 0.5%) with 92% targeting efficiency, enhancing irinotecan tumor penetration 3.2-fold while reducing intestinal toxicity from 28% to 9%.^184^ ^,^ ^185^ Integration of AI across the probiotic research pipeline substantially accelerates therapeutic development and optimization.

Although personalized therapy based on the microbiome holds tremendous promise, its clinical translation faces significant practical challenges. First, regulatory pathways remain unclear. Defining and approving a “drug” composed of live bacteria or bacterial components involves standards far more complex than those for traditional chemical drugs. Second, cost-effectiveness and accessibility pose significant concerns. Highly personalized treatment regimens may incur substantial expenses, limiting their widespread adoption among patients. Finally, long-term safety data remains insufficient. The prolonged side effects of many novel therapies—such as engineered bacteria—are still unknown, necessitating extended monitoring. Consequently, while these cutting-edge technologies demonstrate immense theoretical potential, their journey from laboratory to clinical application requires overcoming multiple scientific, technical, and policy barriers.

Beyond AI-integrated microbiota modulation approaches, diverse therapeutic strategies for gut microbiome manipulation are currently deployed in both clinical practice and experimental investigations. Each modality exhibits distinct therapeutic advantages and constraints, often functioning synergistically in clinical implementation (Table 4).

**Table 4.** Comparison of common gut microbiota modulation techniques.

Modulation techniques	Mechanism	Advantages	Limitations	References
Fecal microbiota transplantation(FMT)	Restoration of gut microbiota by transplanting fecal microbiota from a healthy donor	Demonstrates significant therapeutic efficacy with rapid amelioration of gut dysbiosis.	To mitigate safety risks, including pathogen transmission, standardized protocols are needed for donor-recipient screening, stool preparation, and transplantation timing and procedures.	[186,187]
Precision probiotic therapy	Modulation of gut microbiota through supplementation with specific probiotics	Utilizes AI-driven screening to identify probiotics with anti-tumor potential, enabling the precise design of probiotic combinations for enhanced therapeutic outcomes.	Individualized probiotic strain selection is necessary.	[188,189]
Prebiotics	Indirect modulation of gut microbiota structure by providing nutrients to support beneficial bacteria	Selectively stimulates the growth of beneficial bacteria, increasing gut microbiota diversity.	Significant inter-individual variability in treatment response exists.	[188]
Nanotherapy	Targeted modulation of gut microbiota via delivery of drugs or bioactive molecules to specific intestinal sites using nanomaterials	High target specificity enhances drug delivery efficiency and reduces adverse effects.	Further research is required to fully elucidate safety and efficacy.	[184,185]
Natural extracts and traditional Chinese medicine	Modulation of gut microbiota using plant extracts and traditional Chinese medicine formulations	Features broad source availability and multiple bioactivities.	The majority of research is currently preclinical (animal models), limiting clinical applicability.	[190,191]
Dietary modulation	Influencing gut microbiota composition through dietary modification	Simple, readily implementable, and cost-effective.	Significant inter-individual variability in treatment response exists.	[23,32,192,193]
Phage therapy	Targeted killing of specific bacteria using bacteriophages	High target specificity minimizes disruption of beneficial microbiota.	The technology remains under development and investigation.	[194]
Gene editing	Targeted modification of gut microbiota using gene editing tools	Precisely eliminates antibiotic-resistant bacteria while minimizing interference with beneficial microbiota.	Further research is necessary to fully establish safety and efficacy.	[195]

### 4.3. Drug development

CRC remains a predominant contributor to global cancer mortality, with its pathogenesis intrinsically linked to gut microbiome homeostasis. As a key component of the tumor microenvironment, the gut microbiome coordinates microenvironmental remodeling through metabolic programming, immune regulation, and epigenetic modifications.^55^ ^,^ ^196-199^ Despite these insights, the precise molecular mechanisms mediating microbiota-driven CRC progression remain incompletely understood, impeding the establishment of microbiota-targeted treatment strategies. Through integration of multiomics datasets and text mining approaches, investigators identified 518 microbiota-associated candidate genes, subsequently refined to 48 core differentially expressed genes in CRC utilizing the cancer genome Atlas (TCGA) database. Network analysis of drug‒gene interactions revealed 24 potential therapeutic compounds, establishing novel avenues for precision medicine approaches in CRC.^200^

**Figure 6.**
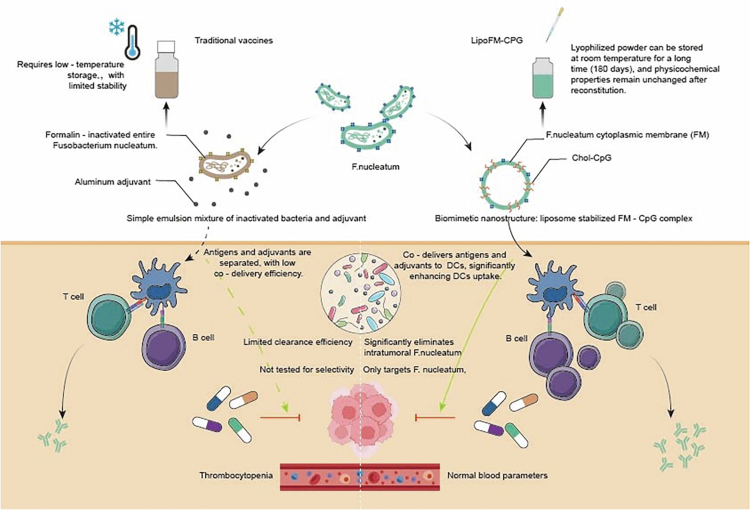
Comparison of nano-vaccine LipoFM-CPG vs. traditional vaccines: traditional vaccine (Left): consists of a conventional formulation of formalin-inactivated whole bacterial cells and alum adjuvant. The system requires strict cold-chain storage conditions with limited stability parameters. The physical separation between antigen and adjuvant components results in suboptimal codelivery efficiency. The formulation demonstrates limited clearance efficacy without validated selectivity, potentially inducing adverse effects, including thrombocytopenia.LipoFM-CPG Nano-Vaccine (Right): Incorporates an advanced biomimetic nanostructure constructed from *Fn* cytoplasmic membranes (FM) and cholesterol-modified CpG (Chol-CpG). The lyophilized formulation demonstrates sustained stability at ambient temperature for 180 d, maintaining intact properties upon reconstitution. The platform facilitates enhanced codelivery of antigen/adjuvant to dendritic cells, substantially improving cellular uptake and selectively eliminating intratumoral *Fn* while maintaining normal hematological parameters and microbiome homeostasis. This innovative nanoplatform addresses conventional limitations through biomimetic nanotechnology, achieving simultaneous targeted bactericidal activity, extended stability, and optimal biosafety profiles.

*Fn*, an anaerobic pathogen from the oral environment, is significantly enriched in the tumor tissues of CRC patients. Beyond its known proinflammatory and immune evasion properties, *Fn* also plays a crucial role in promoting chemotherapy resistance in tumors. *Fn* can secrete specific enzymes or metabolites that directly degrade or modify chemotherapeutic agents (such as 5-fluorouracil, 5-FU), reducing their effective concentration within tumor tissues. *Fn* can also bind to E-cadherin on host cells via surface adhesion molecules like FadA, activating the PI3K/AKT and Wnt/β-catenin signaling pathways. Both pathways suppress the expression of proapoptotic proteins (e.g., Bax, Bak) and upregulate antiapoptotic proteins (e.g., Bcl-2, Bcl-xL), ultimately conferring resistance to chemotherapy-induced apoptosis in tumor cells.^84,201–204^ Although traditional broad-spectrum antibiotics can reduce *Fn* load, their nonselective bactericidal action easily disrupts the gut microbiota, inducing side effects such as drug resistance and immunosuppression.^205^ ^,^ ^206^ To address this bottleneck, researchers have developed a nano-vaccine targeting *Fn*, LipoFM-CPG. This vaccine embeds cholesterol-modified CpG oligodeoxynucleotid (CpG) oligonucleotides (Chol-CPG) into *Fn* autologous membrane vesicles, achieving specific clearance of *Fn* within the tumor. LipoFM-CPG shows enhanced effectiveness and safety compared to traditional vaccines in animal studies, potently activating *F.* nucleatum-specific immune responses and inhibiting tumor growth while preserving the commensal microbiota^122^ (Figure 6). In the future, personalized treatment based on patient gut microbiome characteristics (such as *Fn* abundance and *Bacteroides*/Firmicutes ratio) will become a trend. By integrating microbiome data, host genomic information, and drug response profiles using AI models, it is possible to accurately predict patient sensitivity to vaccines, ICIs, or chemotherapy, and thus design dynamically optimized treatment plans.^207^ For example, for patients with high *Fn* loads, combining LipoFM-CPG with PD-1 inhibitors may synergistically enhance anti-tumor immunity; while for cases primarily characterized by an imbalance of probiotics (such as *Bifidobacteria*), prebiotic dietary intervention can be used to modulate microbiota-host interactions and improve the response rate of existing therapies.^208^ In addition, the mechanism by which natural drugs (such as naringenin) regulate ferroptosis pathways and reshape the tumor microenvironment through microbiota metabolism provides new ideas for multitarget therapy of CRC.^209^

## 5. Application prospects

### 5.1. Clinical application potential

#### 5.1.1. Treatment efficacy and safety enhancement

AI, through the integration of multimodal data including genomic, imaging, and microbiome information,^210^ can substantially enhance both the precision and safety of cancer treatment. In terms of precision improvement, AI enables multidimensional therapeutic target identification by analyzing the complex interaction network between the gut microbiome and cancer genomics. For example, for CRC patients, DL models, by integrating metagenomic and transcriptomic data, successfully identified key gut microbiota biomarkers associated with KRAS mutations, achieving a 91% accuracy rate (sensitivity 88%, specificity 93%) in predicting KRAS mutation status.^211^ Similarly, ML models (such as random forest and XGBoost) built on 16S rRNA sequencing data can distinguish the microbiota characteristics of CRC patients from healthy individuals, with an AUC of 0.93 in the validation cohort and an improved sensitivity of 79% for the detection of early adenomas.^212^

In terms of drug safety, AI can not only screen for potentially effective anticancer drugs^200^ but also predict the efficacy and toxicity risks of existing drugs, dynamically integrating prediction results with clinical practice to optimize treatment plans.^213^ ^,^ ^214^ For example, the COLOXIS model, developed based on ML, analyzes the transcriptomic and microbiomic characteristics of early-stage colon cancer patients to accurately identify oxaliplatin-benefiting individuals (COLOXIS+). The model achieves an AUC of 0.87 for predicting sensitivity, significantly reducing the proportion of ineffective drug use (HR = 0.62, *P* < 0.001), while simultaneously reducing the occurrence rate of grade 3 or above diarrhea and other toxic events from 22% to 8%.^215^

#### 5.1.2. Dynamic treatment optimization

**Figure 7.**
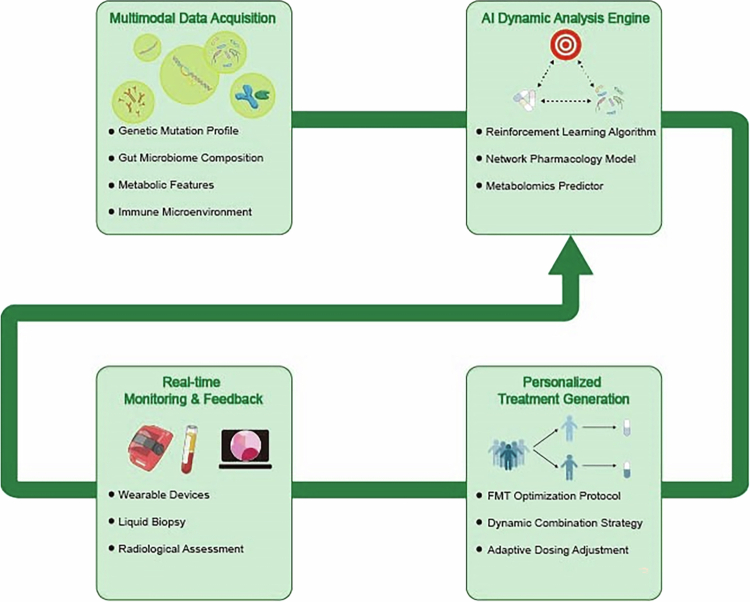
AI-driven process for dynamic optimization of precision tumor treatment: This process mainly contains four core steps: first, multimodal data acquisition integrates genetic mutation, gut microbiome, metabolic features, and immune microenvironment data. Second, AI enables dynamic analysis through FMT donor optimization via RL algorithms, drug‒microbiome interaction prediction, and chemotherapy sensitivity evaluation. Third, the system generates personalized interventions, including optimized FMT protocols, immune combination therapy strategies, and real-time dose adjustment recommendations. Finally, wearable devices, liquid biopsy, and imaging modalities track therapeutic efficacy, with data transmitted back to the AI engine in real-time for continuous treatment optimization.

Based on individual patient differences (such as gene mutations, microbiome composition, and metabolic characteristics), AI can generate highly personalized treatment strategies (Figure 7). For example, the modulating effect of FMT on the effects of ICIs shows significant variability: some clinical trials show that FMT can increase ICI response rates and prolong progression-free survival, while other studies have not observed significant benefits or have reported toxicity related to microbiota dysbiosis. This discrepancy may stem from the multidimensional regulatory mechanisms involved in the effect of the intestinal microbiota on ICI effects, including diversity, abundance of specific microbiota (such as the *Bacteroidetes*/Firmicutes ratio), and metabolites (SCFAs, tryptophan derivatives).^186^ ^,^ ^216^ Through RL algorithms, AI systems dynamically assess functional compatibility between recipient baseline microbiota modules (e.g., butyrate synthesis pathways) and donor metabolic profiles, optimize donor selection, and predict colonization efficacy of key commensals (including *Akkermansia muciniphila* and *Bifidobacteria*), thereby enhancing FMT precision and clinical translation.^217^

Furthermore, multimodal data fusion technology further expands the boundaries of personalized treatment. DL-based metabolomics models can predict the chemotherapy sensitivity and prognostic risk stratification of gastric cancer patients through blood/tissue metabolite characteristics (such as lactate, ketone bodies, and polyamines)^216^; while network pharmacology frameworks can integrate tumor genomic mutations (such as TP53, KRAS), immune microenvironment characteristics (PD-L1 expression, T cell infiltration score), and microbiome data to generate dynamic treatment plan recommendations (such as sequential strategies combining ICIs with targeted drugs).^218^ ^,^ ^219^ Cancer genomic ecosystems such as SCRUM-MONSTAR have implemented AI-driven real-time treatment decision support, optimizing clinical pathways through continuous learning of cross-omics data (whole-exome sequencing, single-cell transcriptomics, microbial metagenomics), and promoting the evolution of precision oncology towards an “adaptive therapy” paradigm.^220^

#### 5.1.3. Microbiota-directed nutritional intervention

The intestinal microbiome demonstrates a significant correlation with therapeutic response in oncology.^8^ Dietary composition, a critical exogenous determinant of gut microbiota, modulates both microbial diversity (exemplified by *Firmicutes*/*Bacteroidetes* ratios) and functional capacities (including polysaccharide utilization and tryptophan metabolism), while influencing tumor progression through metabolite-mediated immunomodulation.^23^ ^,^ ^207^ ^,^ ^221^ ^,^ ^222^ High-fiber dietary interventions augment anti-PD-1 immunotherapy efficacy through enrichment of butyrate-producing bacteria (notably Roseburia spp. and Faecalibacterium prausnitzii), whereas high-fat diets promote proinflammatory microbiota proliferation and attenuate antitumor immunity.^223^ ^,^ ^224^ Clinical investigations demonstrate that AI-guided personalized dietary interventions surpass conventional low-FODMAP approaches in optimizing microbiota homeostasis (achieving a 1.8-fold increase in *α*-diversity) and mitigating therapy-associated gastrointestinal toxicity.^225^ In the future, combining real-time microbiome monitoring (such as fecal metagenomic sequencing) with RL algorithms, AI will be able to generate personalized dietary plans to regulate microbiome balance by analyzing patients' metabolome, microbiome composition, and dietary habits.

### 5.2. Public health significance

AI demonstrates significant advantages in early cancer screening and has been used in the prevention and detection of hepatocellular carcinoma,^226^ lung cancer,^227^ upper gastrointestinal cancers,^228^ and colon cancer.^229^ In drug development, AI accelerates the screening of candidate drugs^200^ and clinical trial design, shortening the new drug development cycle. AI can also assist healthcare workers in diagnosis, reducing the misdiagnosis rate of cancer,^229^ and rapidly predicting new cancer therapeutic drugs.^200^ Public health economic analyzes show that the comprehensive penetration of AI can produce significant cost-effectiveness, having important public health significance. In the future, by building multicenter AI collaboration networks across institutions (such as integrating electronic health records and regional cancer registries), the response efficiency of cancer prevention and control systems can be further enhanced.

## 6. Challenges

### 6.1. Technological challenges

Although contemporary investigations are primarily based on correlation assessment, our understanding of the causal links between the microbiome and tumors remains largely ambiguous. AI-facilitated integration of multiomics datasets holds promise for delineating underlying molecular mechanisms. The intricate host‒microbiome interaction network encompasses multiple biological layers, spanning microbiome, metabolome, genome, and transcriptome domains.^35^ These heterogeneous data matrices present unprecedented complexity in both dimensionality and scale. The comprehensive and efficient integration and analysis of these multiomics datasets to decipher the complex microbiome‒tumor interplay represents a critical methodological challenge requiring urgent attention.

A major challenge accompanying massive data is its heterogeneity. This heterogeneity arises from multiple levels: sample collection (e.g., storage duration, temperature, and transport conditions of fecal samples), DNA extraction (differences in genomic DNA recovery rates and purity due to varying kits and operators), sequencing platforms (Illumina, PacBio, Oxford Nanopore, etc., exhibit systematic biases in read length, error rates, and coverage) and bioinformatics analysis workflows (e.g., differing quality control standards, species annotation databases, OTU clustering parameters). Collectively, these factors contribute to the so-called “batch effect”—systematic measurement deviations in the same biological sample under different experimental conditions.

This heterogeneity poses a fundamental obstacle to the clinical translation of AI models, primarily manifesting as a dual erosion of model generalizability and research reproducibility. First, regarding generalizability, models trained on specific cohorts (e.g., Cohort A) often overfit to cohort-specific technical characteristics (such as microbial composition biases introduced by DNA extraction kits used in a particular laboratory). When directly transferred to independent cohorts from different experimental conditions (e.g., Cohort B), the model's predictive performance typically plummets—not because the biological signal is lost, but because the model misinterprets “technical noise” as discriminative features. For instance, a microbial classifier trained on a specific DNA extraction method in one laboratory may completely fail when confronted with data from laboratories using different extraction kits.^230^ Second, regarding reproducibility, data heterogeneity is the root cause of difficult-to-replicate research results.^231^ Different research teams studying the same disease (e.g., colorectal cancer) may report vastly divergent “core” microbial biomarkers, largely attributable to the aforementioned heterogeneity in experimental workflows and population cohorts. When a biomarker feature identified by an AI model fails to replicate in independent, heterogeneous cohorts, the credibility of its scientific discovery is called into question. This “hockey stick curve” performance decay—where models excel on training and internal validation sets but suffer steep declines in independent external validation—highlights the reproducibility crisis plaguing current microbiome AI research and represents a core obstacle to clinical translation. Therefore, unless data heterogeneity is systematically identified, quantified, and corrected, even AI models demonstrating strong performance in internal validation will face severe limitations in their clinical translation potential.

Despite AI's superior analytical and predictive capabilities, model interpretability and reliability remain fundamental challenges in clinical implementation.^232^ ^,^ ^233^ In microbiome-targeted precision medicine, AI frameworks must demonstrate transparent decision-making processes, enabling clinicians and investigators to comprehend the underlying algorithmic rationale and computational logic. Contemporary sophisticated AI architectures frequently operate as “black boxes,” obscuring their internal computational mechanisms and decision pathways. Model robustness and reliability assume critical importance in clinical deployment, particularly in complex biomedical analytics, necessitating comprehensive validation through rigorous statistical frameworks to ensure computational accuracy and stability.^234^ This validation paradigm demands not only substantial training and testing datasets but also extensive cross-validation across diverse clinical contexts to establish model generalizability.

Furthermore, the issue of reproducibility in bioinformatics analysis workflows represents another critical dimension of technical challenges. Process heterogeneity not only leads to conflicting biomarker identifications but also directly undermines the reliability of AI models built upon these markers. If microbial features used for model training inherently depend on specific, nonstandardized analysis workflows, the model's generalization capability will inevitably suffer when confronted with new data generated by different processes. Therefore, advancing the standardization and transparency of bioinformatics workflows is a necessary prerequisite for constructing robust AI models suitable for clinical deployment.

### 6.2. Ethical and legal challenges

#### 6.2.1. Algorithmic bias

The issue of algorithmic fairness—specifically, disparities in model performance across different populations—has evolved from a theoretical risk into a tangible challenge. For instance, a multicenter study on colorectal cancer screening revealed that AI classifiers trained on gut microbiome data from European and American populations exhibited a sharp drop in specificity from 92% to 67% when applied to Asian and African cohorts. Further analysis revealed this performance degradation stemmed not from technical failure, but from the training data's lack of representation for microbial diversity in nonwhite populations, such as Prevotella abundance.^143^ This case reveals a profound ethical crisis: when AI models are primarily trained on data from dominant populations, minority or low-income groups may be excluded from precision medicine due to low model accuracy, thereby exacerbating rather than reducing existing health inequalities. Consequently, addressing algorithmic bias is not merely a technical issue but a matter of justice concerning healthcare equity. Before clinical deployment, models must undergo rigorous multicenter, multipopulation fairness audits to ensure consistent performance across diverse subgroups.

#### 6.2.2. Data governance

Even with the widespread application of AI in daily life, privacy protection remains a recurring issue. In the medical field, in particular, confidentiality of patients' protection and information security are crucial ethical considerations. In healthcare contexts, confidentiality of patients' safeguards and information security protocols represent critical ethical imperatives. Patient biomedical profiles, encompassing microbiome compositional and functional metadata, constitute highly sensitive protected health information requiring stringent privacy and security measures. Maintaining patient privacy throughout data acquisition, storage, and utilization represents an essential responsibility for investigators in this domain.^235^ Moreover, escalating cybersecurity threats and data breaches necessitate robust protective mechanisms to prevent unauthorized access and manipulation of sensitive biomedical data. However, traditional “deidentification” processing may no longer suffice in the era of microbiome data. A cautionary case is the “OKCupid” data reidentification incident: although user data had been stripped of names and ID numbers upon release, researchers successfully reidentified some individuals by cross-referencing publicly available microbiome data with social media metadata.^235^ This implies that even after removing direct identifiers, the uniqueness of gut microbiome data—akin to a human “microbial fingerprint”—can still serve as a trail for tracing personal identity, lifestyle habits, and even geographic location.

#### 6.2.3. Patient informed consent

Clinical implementation of AI technologies necessitates comprehensive ethical oversight and regulatory compliance.^235^ In microbiome-targeted precision medicine, ethical frameworks must address multiple domains, encompassing patient-informed consent protocols, data utilization parameters, and risk-benefit assessments. Consequently, regulatory authorities must establish robust governance frameworks to ensure appropriate deployment of AI technologies. This multidisciplinary endeavor mandates coordination among technical specialists, bioethicists, legal scholars, and patient advocates to establish comprehensive regulatory guidelines.^236^

However, the traditional informed consent model faces severe challenges in the AI era. A cautionary case is the 2017 data-sharing incident between the Royal Free NHS Trust in the UK and Google DeepMind: to develop an AI early warning system for acute kidney injury, the trust provided DeepMind with identifiable medical data from 1.6 million patients without adequately informing them that their data would be used for AI testing.^237^ The UK Information Commissioner's Office ultimately ruled this violated data protection laws, as patients had “no reasonable expectation” of such secondary data usage, and the scale of data sharing far exceeded what was necessary for development and testing. This case reveals a core contradiction: AI model development requires large-scale, diverse data for training and iteration, yet the “one-time” informed consent signed by patients for specific purposes cannot cover the “dynamic” uses of data in subsequent model optimization.

In summary, to navigate ethical minefields, AI-driven precision medicine for the microbiome must transition from principled advocacy to concrete governance. This requires establishing multicenter, multipopulation standards for fairness validation, developing privacy-preserving computing technologies for microbiome data, and collaborating with regulators to explore new informed consent paradigms adapted to dynamic AI models.

### 6.3. Challenges in clinical application

While AI-enabled personalized therapeutic strategies have demonstrated preliminary efficacy in experimental settings, the clinical translation of these interventions requires systematic protocol standardization and rigorous validation through controlled clinical trials to establish safety profiles and therapeutic efficacy, representing a complex trajectory in therapeutic development.

**Figure 8.**
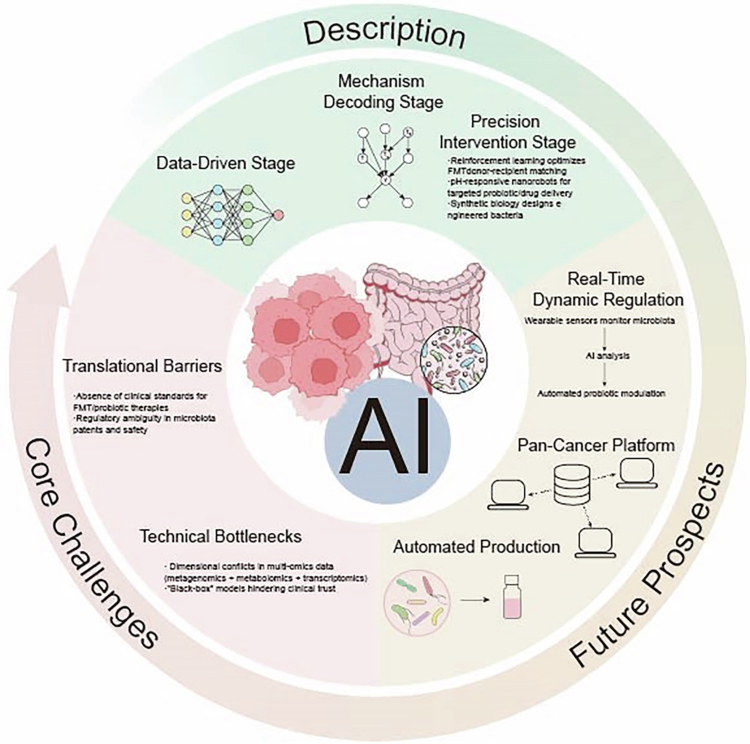
Development history of AI-regulated gut microbiota against tumors: in the initial stage, ML approaches enable the systematic screening of microbial biomarkers associated with CRC and integrate multiple omics datasets, including metagenomic and metabolomic data, through DL algorithms. Subsequently, AI-based causal inference analysis elucidates the underlying microbial mechanisms. The field is currently advancing into the precision intervention stage, where RL algorithms optimize FMT donor selection, pH-sensitive nanorobotic systems facilitate precise targeted delivery of therapeutic agents, and synthetic biological approaches enable the development of engineered bacterial strains that secrete antitumor metabolites. Future developments will incorporate wearable biosensors for real-time microbiota monitoring and automated AI-based regulation, while establishing integrated cross-cancer databases to facilitate automated production of personalized microbial therapeutics in specialized microbiome manufacturing facilities. However, significant challenges remain, including the complexity of multidimensional omics data integration, the limited interpretability of AI models, the absence of standardized clinical protocols, and unresolved ethical and regulatory considerations, which collectively impede clinical translation.

Clinical adoption of AI technologies faces substantial challenges regarding healthcare practitioner acceptance and competency development in novel methodologies. The primary bottleneck in AI adoption stems from barriers to trust and understanding among physicians. Complex deep learning models often produce predictions as “black boxes,” failing to provide biologically grounded outputs that clinicians can comprehend and trace. This lack of interpretability fundamentally conflicts with physicians' clinical decision-making patterns, which are derived from pathophysiological mechanisms. Healthcare practitioners often prioritize experiential clinical judgment over AI-driven insights, reflecting a knowledge‒trust gap that potentially impedes technological integration and clinical implementation.^238^ Consequently, enhanced professional development programs are imperative to cultivate technological literacy and algorithmic understanding among healthcare practitioners^87^ (Figure 8).

Furthermore, clinical environments are dynamic, whereas AI models are typically static. Changes in laboratory conditions, reagent batches, sequencing technology iterations, and even shifts in dietary habits among populations can all cause subtle variations in the distribution of microbiome data—a phenomenon known as model drift.^156^ A model trained and validated 5 y ago may exhibit undetected performance degradation when applied to today's data. Therefore, AI systems deployed in clinical settings require continuous monitoring and active learning mechanisms capable of automatically detecting performance decline and updating themselves upon exposure to new data.

Moreover, there is currently no clear regulatory framework for “AI+microbiome diagnostics/intervention” products. Such products may simultaneously fall under in vitro diagnostic reagents, laboratory-developed tests, and software-as-a-medical-device (SaMD) categories, facing fragmented oversight or regulatory gaps. More critically, the absence of unified clinical validation standards—such as minimum required validation cohort sizes, multicenter external validation requirements, and batch effect control protocols—hinders the comparability of research findings, thereby impeding regulatory approvals and health insurance coverage.^239^

## 7. Discussion

As a vital component of the human body, the gut microbiome not only plays a fundamental role in sustaining human health,^240^ but also shows strong associations with tumor onset, progression, and treatment outcomes. Its influence operates through multiple pathways: modulating host immune responses, impacting metabolic processes, and generating bioactive metabolites.^196^ ^,^ ^241^ With its complex and variable nature, the gut microbiome's effect on tumors creates significant challenges for established research methods attempting to thoroughly examine its network of influences.^242^ Traditional culture methods and single-omics analyzes often only capture fragments of this complex ecosystem, failing to reveal the global relationships with the microbiome and between the microbiome and the host. AI technology, with its powerful data integration and pattern recognition capabilities, provides new tools for elucidating the mechanisms of microbiome–host–tumor interactions and is being widely applied in various aspects of gut microbiome and tumor research. With AI tools, we can not only improve the accuracy of tumor diagnosis but also predict treatment efficacy and adverse reactions after treatment. The efficiency of experimental data collection, processing, and related mechanism analysis can also be significantly improved with the assistance of AI. Furthermore, based on AI development and the elucidation of processes by which the intestinal microbiome influences tumors, personalized targeted cancer therapy is emerging as a new trend, particularly showing significant clinical application potential in CRC and other gastrointestinal tumors.

This article focuses on the targeted analysis of the interaction between the gut microbiome and the tumor immune microenvironment using AI technology. By integrating multiomics data (metagenomics, metabolomics, transcriptomics) using ML models (such as random forests and XGBoost), it is possible to accurately predict and identify microbiome biomarkers associated with ICI efficacy.^146^ The application of CNNs for microbiome structure classification and tumor detection, combined with RNNs for forecasting intestinal microbiome alterations, reveals AI's effectiveness in processing massive data collections. These technologies not only break through the limitations of traditional single-dimensional research but also provide theoretical support for personalized treatment, constructing a predictive chain from microbiome characteristics to host immune phenotype and then to treatment response. Based on the rapid development of these foundational technologies, the development of new therapeutic drugs and targeted therapies has also progressed rapidly.

Personalized targeted modulation is an important direction for future cancer treatment. Multiomics integration technology based on AI can identify patient-specific microbiome characteristics (such as inhibition of the butyrate metabolism pathway or increased secondary bile acids), thereby designing dynamic treatment plans. AI-assisted precision probiotic combinations and targeted drug screening provide new strategies for clinical application. In the future, combining real-time microbiome monitoring with RL algorithms, AI is expected to achieve dynamic optimization of treatment plans. Through personalized targeted modulation, it will improve efficacy and reduce toxicity, thereby providing patients with a more precise and effective treatment experience. From microbiome intervention and prevention in high-risk populations, to real-time monitoring and adjustment during treatment, and to prognosis and recurrence prediction, AI-driven microbiome management will permeate the entire process of tumor management, significantly accelerating human research and treatment of tumors, ultimately leading to conquering cancer.

Despite the immense potential of AI technology, this field still faces multiple challenges. At present, the causal relationship between the microbiota and tumors remains unclear, with most current research still confined to the level of correlation. While *Fn* is associated with CRC chemotherapy resistance, the molecular details of its oncogenic mechanism (such as Wnt/β-catenin pathway activation) still require further validation. Future work requires more in vivo experiments (such as gene-edited strains and germ-free animal models). Utilizing advanced techniques like gene-edited strains, germ-free/syngeneic animal models, and organoid coculture, we will functionally validate key microbiota‒host interaction nodes identified by AI to establish clear causal chains. This will provide a solid scientific foundation for precision interventions.

Secondly, the “black box” nature of models limits clinical trust. The lack of interpretability in AI model decision-making processes makes it difficult for physicians to understand the basis for their predictions, hindering their application in critical clinical decisions. Moving forward, significant efforts must be made to develop and apply explainable AI technologies. This will transform the model's “black box” decisions into biological logic comprehensible to clinicians, thereby enhancing reliability and acceptance in diagnostic and therapeutic practices.

Additionally, data quality and standardization are also major obstacles. Differences in sample processing, sequencing methods, and data analysis workflows across different studies make it difficult to directly compare and integrate results. Moreover, existing studies largely rely on mouse models or in vitro gut-on-a-chip systems, but these models cannot fully simulate the heterogeneity and dynamism of the human gut microenvironment, leading to difficulties in clinical translation of some findings. To address data heterogeneity, researchers are adopting multiple strategies: First, employing batch correction methods (such as the ComBat algorithm) to eliminate batch effects; second, utilizing multimodal learning frameworks to automatically align different data sources via deep neural networks, thereby reducing reliance on data homogeneity; third, applying transfer learning by pretraining models on large-scale source cohorts and then fine-tuning them to target cohorts to enhance performance on small samples and mitigate batch bias; Fourth, promoting standardized experimental protocols and establishing public reference databases to reduce heterogeneity at its source. Moving forward, developing AI algorithms capable of robustly processing heterogeneous data will be pivotal for achieving clinical translation in microbiome research.

Furthermore, in terms of clinical application, while novel therapies targeting the microbiota (such as the *Fn*-specific nanoparticle vaccine LipoFM-CPG) have demonstrated potential in preclinical studies, large-scale clinical trials, long-term safety, and scalable production remain critical hurdles to overcome. Therefore, rigorous randomized controlled trials should be designed for AI-guided personalized microbiota intervention strategies (e.g., FMT donor matching based on the GEI, precision probiotic combinations, or targeted nanomedicines). These trials must clearly define inclusion/exclusion criteria, intervention protocols, and endpoint measures to systematically evaluate their clinical value in enhancing efficacy, reducing toxicity, and dynamically optimizing treatment regimens.

Through examining the intricate system of the gut microbiome, AI technology has established a novel approach to cancer therapy: the integrated “microbiome–immune–metabolic” intervention. This paradigm transcends the traditional single-target approach, emphasizing the modulation of the microbiome as a pivotal node to simultaneously influence both immunity and metabolism, two core pillars in cancer development. However, further challenges need to be addressed, including data integration, model interpretability, and ethical regulation. This requires the establishment of more comprehensive cross-center, multicohort big data platforms and unified data standards, the development of explainable AI technologies to enhance model transparency, and the formulation of ethical guidelines addressing the privacy of microbiome data and the safety of interventions. Future research should focus on establishing organoid models that more closely mimic human physiology, promoting clinical trials targeting the microbiome, and exploring the cross-integration of AI with synthetic biology and gene editing technologies. With technological maturation, personalized microbiome modulation is expected to become a core pillar of precision oncology, significantly improving patient prognosis and quality of life, and enabling more individualized, effective, and less toxic comprehensive treatments.

## Supplementary Material

Supplementary Material Supplementary_Table.docx

## Data Availability

This systematic review is based on previously published studies and publicly available data. All data analyzed in this study were obtained from peer-reviewed articles, clinical trials, and scientific databases as cited in the manuscript. No new data were generated or collected for this review. Further inquiries can be directed to the corresponding author.

## Abbreviations

AI: Artificial Intelligence
ANN: Artificial Neural Network
ASV: Amplicon Sequence Variant
AUC: Area Under the Curve
BSI: Bloodstream Infection
CagA: Cytotoxin-associated gene A
CDCA: Chenodeoxycholic Acid
CNN: Convolutional Neural Network
CNS: Central Nervous System
COLOXIS: Colon Oxaliplatin Characteristics
CpG: CpG Oligodeoxynucleotid
CRC: Colorectal Cancer
CTLA-4: Cytotoxic T-lymphocyte-associated protein 4
DL: Deep Learning
DNA: Deoxyribonucleic Acid
EMT: Epithelial-Mesenchymal Transition
FadA: Fusobacterium Adhesion A
FL: Federated Learning
FM: Fusobacterium nucleatum Cytoplasmic Membranes
FMT: Fecal Microbiota Transplantation
Fn: Fusobacterium nucleatum
FXR: Farnesoid X Receptor
GAN: Generative Adversarial Network
GEI: Gut Ecology Index
GMSM: Gut Microbiota-Serum Metabolome
GPR41/43: G Protein-Coupled Receptor 41/43
GRU: Gated Recurrent Unit
HATs: Histone Acetyltransferases
HCA: Hierarchical Clustering Analysis
HDACs: Histone Deacetylases
HR: Hazard Ratio
IA: Intracranial Aneurysm
IBD: Inflammatory Bowel Disease
ICI: Immune Checkpoint Inhibitor
IFN-γ: Interferon-gamma
IgA: Immunoglobulin A
IL-1β: Interleukin-1 beta
IL-6/10: Interleukin-6/10
IS: Indoxyl Sulfate
LDA: Linear Discriminant Analysis
LPS: Lipopolysaccharide
LSTM: Long Short-Term Memory
MDP: Markov Decision Process
ML: Machine Learning
MMP: Matrix Metalloproteinase
mPFS: Median Progression-Free Survival
MSI: Microsatellite Instability
MSS: MicroSatellite Stable
NF-κB: Nuclear Factor Kappa B
ORR: Objective Response Rate
OUT: Operational Taxonomic Unit
PCA: Principal Component Analysis
PD-1/PD-L1: Programmed Death-1/Ligand-1
pH: Potential of Hydrogen
PVT: Portal Vein Thrombosis
RIA: Ruptured Intracranial Aneurysm
RL: Reinforcement Learning
RNN: Recurrent Neural Network
ROS: Reactive Oxygen Species
SaMD: Software-as-a-Medical-Device
SCFA: Short-Chain Fatty Acid
sNMF: Supervised Non-negative Matrix Factorization
SOM: Self-Organizing Feature Mapping neural network algorithm
STAT3: Signal Transducer and Activator of Transcription 3
SVM: Support Vector Machine
TCGA: The Cancer Genome Atlas
TCS: Triclosan
TDA: Topological Data Analysis
TGR5: Takeda G protein-coupled receptor 5
TL: Transfer Learning
TLR4: Toll-Like Receptor 4
TNF-α: Tumor Necrosis Factor-alpha
UIA: Unruptured Intracranial Aneurysm
XGBoost: eXtreme Gradient Boosting
