# Supplementary material for: Decoding the microbiome: artificial intelligence-targeted gut microenvironment breakthroughs in personalized cancer therapy
Source: Gut Microbes. 2026 May 29;18(1):2672791. doi: 10.1080/19490976.2026.2672791 (PMC13224768; doi:10.1080/19490976.2026.2672791)
Supplement: Supplementary Material — Supplementary_Table.docx [file KGMI_A_2672791_SM5794.docx]

**Table 1** Cancer-Associated Gut Bacteria: Mechanisms & Therapeutic Potential

| Sites | Cancer-associated bacteria | Mechanisms | Potential for Therapy |
| --- | --- | --- | --- |
| Esophagus | *Fusobacterium nucleatum*^1^ | Pro-inflammatory cytokine release, suppressing anti-tumor immune responses. | High (Preclinical evidence in GI cancers; targeting Fn adhesion/invasion may enhance immunotherapy) |
|  | *Porphyromonas gingivalis*^2^ | Activation of the TGFβ-SMAD/YAP/TAZ signaling pathway, providing a suitable tumor microenvironment. | Medium (Associative evidence; therapeutic relevance not yet demonstrated in vivo) |
| Stomach | *Dialister pneumosintes*^3^ | Altered metabolite production modifying the gastric mucosal environment. | Low (Mechanism unclear; limited therapeutic evidence) |
|  | *Helicobacter pylori*^4,5^ | Group 1 Carcinogen: CagA protein induces epithelial damage, triggering gastric inflammation. | High (Established therapeutic target; eradication therapy reduces cancer risk) |
|  | *Parvimonas micra*^6^ | Promotion of matrix metalloproteinase (MMP) secretion, accelerating tumor invasion. | Low (Co-occurs with oncogenic bacteria; direct therapeutic evidence lacking) |
|  | *Peptostreptococcus stomatis*^6^ | Activation of the IL-6/STAT3 pathway, inhibiting apoptosis. | Medium (Associated with IL-6/STAT3 activation; bacterial targeting not yet validated) |
|  | *Slackia exigua*^7^ | Bile acid metabolites promote cell proliferation. | Low (Limited evidence for direct therapeutic targeting) |
|  | *Streptococcus anginosus*^6,8^ | Reactive oxygen species (ROS) production inducing gene mutations. | Low (ROS production implicated; no specific anti-bacterial strategy developed) |
| Colon and Rectum | *Bacteroides fragilis*^9^ | Secretion of B. fragilis toxin ( BFT ) disrupting the intestinal barrier and activating the NF-κB inflammatory pathway. | High (Targeting BFT toxin or NF-κB pathway suppresses tumorigenesis in preclinical models) |
|  | *Escherichia coli*^10,11^ | Production of colibactin toxin inducing DNA double-strand breaks, affecting DNA repair, promoting tumor cell proliferation. | High (Colibactin inhibitors under development; potential for chemoprevention) |
|  | *Enterococcus faecalis*^12^ | Superoxide production leading to chromosomal instability. | Medium (Associated with oxidative DNA damage; specific targeting not established) |
|  | *Fusobacterium nucleatum*^13,14^ | Key bacteria: Disruption of the intestinal epithelial barrier, damage to cellular DNA, activation of oncogenic signaling pathways, promotion of tumor cell metastasis, and promotion of inflammatory responses. | High (Multiple therapeutic strategies: antibiotics, immunotherapy, Fn-targeted vaccines) |
|  | *Peptostreptococcus stomatis*^15^ | Synergistic promotion of tumor growth with *Fn* . | Low (Frequently co-occurs with Fn; therapeutic value uncertain) |
| Pancreas | *Acinetobacter*^16^ | Activation of the TLR4 pathway promoting fibrosis. | Low (Limited therapeutic evidence; mainly as a biomarker) |
|  | *Helicobacter pylori*^17,18^ | Induction of chronic pancreatitis → pancreatic intraepithelial neoplasia. | Low (Epidemiological association inconsistent; therapeutic relevance unproven) |
|  | *Pseudomonadales*^19^ | Promotion of immunosuppressive microenvironment (increased Treg cells). | Low (Taxonomic resolution too broad; functional role unclear) |
| Gallbladder | *Helicobacter pylori*^20^ | Gallstone formation → chronic cholecystitis → carcinogenesis. | Medium (Plausible biological link; clinical benefit not yet demonstrated) |
|  | *Salmonella Typhi*^21,22^ | Risk in chronic carriers: Abnormal bile acid metabolism → gallbladder epithelial hyperplasia. | Medium (Antibiotic eradication may reduce cancer risk in carriers) |
| Bile Ducts | *Helicobacter spp*^23^ | Induction of bile duct epithelial DNA damage and high IL-8 expression. | Medium (Detected in bile; causal role and druggability remain uncertain) |
|  | *Klebsiella pneumoniae*^24^ | Production of β-glucuronidase leading to secondary bile acid accumulation. | Medium (Targeting β-glucuronidase or bile acid metabolism may be therapeutic) |
|  | *Ruminococcus Gnavus group*^24^ | Expression of B-cell superantigens, leading to excessive IgA production and exacerbating inflammatory responses. | Low (Mechanism unclear; therapeutic potential under investigation) |

**Table 2** Core Technical Principles and Applications

| Technical Categories | Core Principles | Strengths | Limitations | Typical Applications |
| --- | --- | --- | --- | --- |
| Deep Learning (DL) | Utilizing deep neural networks to process and analyze large datasets, automatically identifying patterns and solving complex problems.^25,26^ | Automatic feature extraction; well-suited for high-dimensional and complex data; strong end-to-end learning capability. | Requires large amounts of training data; High computational costs; “Black box” characteristics make decision logic difficult to trace. | Classification, functional annotation, and disease prediction of gut microbiota.^27^ |
| Convolutional Neural Networks (CNNs) | Local connectivity and weight sharing extract spatial features, while pooling layers reduce dimensionality.^28^ | Excellent at capturing spatial features; effective for image and structured data; weight sharing reduces overfitting. | Requires a large number of annotated images; sensitive to input dimensions; poor interpretability. | Colony morphology recognition and endoscopic image analysis.^29-31^ |
| Recurrent Neural Networks (RNN) | Processing sequential data, capable of capturing dynamic changes in time series.^32^ | Ideal for sequential modeling; handles variable-length sequences; retains temporal memory of past inputs. | Gradient vanishing problem; slow training speed; difficulty in modeling long-term dependencies. | Simulation and prediction of dynamic changes in gut microbiota.^32^ |
| Supervised Learning (XGBoost) | Gradient boosting framework combined with regularization and second-order Taylor expansion, optimizing the handling of high-dimensional sparse data.^33^ | Highly effective with high-dimensional sparse data; incorporates regularization to prevent overfitting; fast training speed; relatively interpretable. | Limited feature extraction capability; sensitive to changes in data distribution. | CRC diagnosis and detection, prediction of characteristic microorganisms.^34-36^ |
| Reinforcement Learning (RL) | Based on Markov Decision Processes , learning optimal strategies through the interaction of an agent with its environment to maximize cumulative reward.^37,38^ | Suitable for dynamic decision-making problems; adapts to environmental changes; optimizes for long-term cumulative rewards. | Training instability; challenging reward function design; requires extensive trial and error. | Prediction of degradation and biosynthesis pathways of phenolic compounds in the gut microbiome.^39,40^ |
| Federated Learning (FL) | Multiple participants collaboratively train a global model without sharing raw data through encrypted parameter exchange or model updates.^41^ | Preserves data privacy; enables distributed model training; well-suited for multi-center collaborative studies. | High communication costs; Difficult to process heterogeneous data. | Integration of multi-omics data in gut microbiota research, avoiding the export of raw data and protecting privacy.^42^ |
| Transfer Learning (TL) | Finding and leveraging similarities between source and target domains. | Effective in low-data regimes (few-shot learning); accelerates model convergence; enhances cross-domain generalization. | Performance is poor when the source domain and target domain differ significantly. | Overcoming geographical limitations to achieve high-precision diagnosis of cross-regional tumor microbial markers.^43^ |
| Generative Adversarial Networks (GANs) | Generating high-quality data through the adversarial and iterative optimization of two neural networks. | Capable of generating realistic synthetic data; increases dataset diversity; useful for data augmentation and simulation. | Training difficulty is high; potential for model collapse. | Simulation of the abundance distribution of microbial communities.^44^ |

**Table 3** Performance of AI Models in Microbiome-Based Cancer Diagnostics

| Cancer Types | Model Applications: | Data Types | Key Findings | Clinical Utility | Limitations |
| --- | --- | --- | --- | --- | --- |
| Multiple Cancer Types | Multi-class Model | Integrated data from 2320 fecal metagenomes^45^ | Differentiation of 9 disease phenotypes, including CRC. | Enables multi-disease differential diagnosis and early screening. | High model complexity; weak interpretability; requires large-scale validation. |
| CRC | Machine Learning integrating multi-omics data | Metagenomic + Serum Metabolomic data^46^ | Improved specificity in early CRC detection. | Enhances early CRC screening accuracy for high-risk populations. | Limited sample size; metabolic data stability affected by individual variation. |
| CRC | Random Forest Model | Gut microbial characteristics sub-grouping patients^47^ | Significantly improved diagnostic accuracy. | Improves diagnostic accuracy and pathological subtype identification. | Feature selection depends on training data; generalization requires external validation. |
| CRC | SHAP algorithm | Gut microbiome data^48^ | Identification of specific bacteria significantly associated with CRC. | Provides interpretable biomarkers for mechanistic studies and target development. | Correlation does not imply causation; requires experimental validation. |
| CRC | SVM Classifier | Metagenomic data | Improved model prediction accuracy and robustness through recursive feature elimination for gene selection. | Suitable for gene signature screening and high-dimensional data classification. | Sensitive to data distribution; high computational cost. |
| CRC (Poor Prognosis) | Machine Learning Model | Characteristics of Gut Microbiota Dysbiosis (*Bacteroides/Enterococcus* Ratio) | Prediction of bloodstream infection risk after chemotherapy. | Guides preventive interventions and personalized supportive care before chemotherapy. | Single-center cohort; requires prospective multi-center validation. |
| Early-Stage HCC | Random Forest Model | Bacterial metagenomic data | Prediction of precancerous lesions in liver cancer. | Non-invasive early warning for high-risk populations. | Bacterial composition stability affected by diet/environment. |
| Lung Cancer | Machine Learning Model | Specific bacterial markers (such as *Fusobacterium*) | Effective prediction of early lung cancer risk. | Microbiome-based biomarkers supplement imaging diagnostics for unclear cases. | Gut-lung axis mechanism unclear; confounded by smoking and environmental factors. |
| Recurrent Ovarian Cancer | Integrated Analysis (including ML) | Tumor Transcriptomic + Fecal Metabolomic + Gut Microbiome data | Revealed the correlation between gut microbiome and metabolome changes and immunotherapy response. | Multi-omics basis for immunotherapy response prediction and patient stratification. | Small sample size; multi-omics integration methods not standardized; long clinical translation path. |
| Stage II-III Colon Cancer | Machine Learning Model | Gut Microbiome + Transcriptome^49^ | Prediction of therapeutic benefit from oxaliplatin-based adjuvant chemotherapy. | Guides oxaliplatin-based adjuvant chemotherapy selection to maximize treatment benefit. | Microbiome not integrated (needs supplementation); requires prospective trials. |

**Table 4** Comparison of Common Gut Microbiota Modulation Techniques

| Modulation Techniques | Mechanism | Advantages | Limitations | References |
| --- | --- | --- | --- | --- |
| Fecal Microbiota Transplantation(FMT) | Restoration of gut microbiota by transplanting fecal microbiota from a healthy donor | Demonstrates significant therapeutic efficacy with rapid amelioration of gut dysbiosis. | To mitigate safety risks, including pathogen transmission, standardized protocols are needed for donor-recipient screening, stool preparation, and transplantation timing and procedures. | ^50,51^ |
| Precision Probiotic Therapy | Modulation of gut microbiota through supplementation with specific probiotics | Utilizes AI-driven screening to identify probiotics with anti-tumor potential, enabling the precise design of probiotic combinations for enhanced therapeutic outcomes. | Individualized probiotic strain selection is necessary. | ^52,53^ |
| Prebiotics | Indirect modulation of gut microbiota structure by providing nutrients to support beneficial bacteria | Selectively stimulates the growth of beneficial bacteria, increasing gut microbiota diversity. | Significant inter-individual variability in treatment response exists. | ^52^ |
| Nanotherapy | Targeted modulation of gut microbiota via delivery of drugs or bioactive molecules to specific intestinal sites using nanomaterials | High target specificity enhances drug delivery efficiency and reduces adverse effects. | Further research is required to fully elucidate safety and efficacy. | ^54,55^ |
| Natural Extracts and Traditional Chinese Medicine | Modulation of gut microbiota using plant extracts and traditional Chinese medicine formulations | Features broad source availability and multiple bioactivities. | The majority of research is currently pre-clinical (animal models), limiting clinical applicability. | ^56,57^ |
| Dietary Modulation | Influencing gut microbiota composition through dietary modification | Simple, readily implementable, and cost-effective. | Significant inter-individual variability in treatment response exists. | ^58-61^ |
| Phage Therapy | Targeted killing of specific bacteria using bacteriophages | High target specificity minimizes disruption of beneficial microbiota. | The technology remains under development and investigation. | ^62^ |
| Gene Editing | Targeted modification of gut microbiota using gene editing tools | Precisely eliminates antibiotic-resistant bacteria while minimizing interference with beneficial microbiota. | Further research is necessary to fully establish safety and efficacy. | ^63^ |

**Table of Key References**

| Reference Number | Cohort Size | Model Type | Validation Strategy |
| --- | --- | --- | --- |
| 83 | 2,298 participants (multi-center cohorts from China, Austria, Germany, etc.) | Random Forest | External validation (cross-cohort validation, e.g., validating the Chinese cohort model on the German cohort) |
| 85 | 1,073 participants (Chinese population, including healthy controls, prediabetes, and type 2 diabetes) | Random Forest with SHAP interpretability analysis | 10-fold cross-validation |
| 86 | 60 participants (Parkinson's disease patients and healthy controls) | Convolutional Neural Network (CNN) for imaging sensor data analysis | Internal validation (train/test split, e.g., 80/20) |
| 88 | 786 participants (colorectal cancer patients and controls, from two independent Chinese cohorts) | Random Forest + Logistic Regression | External validation (validated the model using an independent cohort) |
| 89 | 75 cancer patients (discovery cohort) + external validation cohort | Machine learning models using bacterial taxa or functional relative abundances | External validation (AUC 78.6-81.1%) |
| 90 | 524 HBV-HCC patients and controls (retrospective discovery cohort: 364 cases, 160 controls) + 215 prospective validation participants (91 cases, 124 controls) | Random Forest | Prospective validation (AUC 0.7971 for oral genera, 0.8084 for gut genera) |
| 91 | Discovery cohort: 106 patients with rare cancers; Validation cohort: 364 patients from six comparable studies | Strain-resolved machine learning models | External validation across six independent cohorts (n=364) |
| 93 | 28 patients with non-Hodgkin lymphoma undergoing HSCT | Machine learning-based BSI risk index | Internal validation (sensitivity 90% at specificity 90%) |
| 95 | 187 UC patients (12,900 EC images for training) + 525 independent segments from 100 patients for validation | Computer-aided diagnosis (CAD) system | Independent validation set (sensitivity 74%, specificity 97%, accuracy 91%) |
| 100 | 332 patients with UC (5,875 endoscopic images + 20 full-length videos) | Convolutional Neural Network (CNN) | Internal validation (compared with endoscopist scoring, achieving 86.54% accuracy) |
| 106 | CTRPv2 public dataset | OmniNet-Fusion (hybrid attention-based CNN-RNN model) | Internal validation (AUC-ROC 0.96, accuracy 94.2%) |
| 107 | Not specified in title (multi-cancer multi-omics dataset) | Interpretable graph Kolmogorov-Arnold networks | Not specified in title |
| 112 | Discovery cohort: 156 participants; Validation cohort: 335 participants from different countries | Metagenomic taxonomic markers | External validation (independent patient and control populations from different countries) |
| 114 | 969 fecal metagenomes (meta-analysis of five public datasets + two new cohorts, validated on two additional cohorts) | Microbiome-based predictive signatures | External validation across multiple independent cohorts (average AUC 0.84) |
| 115 | 112 melanoma patients (fecal microbiome analysis on n=43: 30 responders, 13 nonresponders) | Not applicable (diversity and composition analysis, not predictive model construction) | Not applicable (observational association study) |
| 118 | 1,002 twins and unrelated healthy adults (UK PREDICT 1 study) + 100 US validation cohort | Machine-learning model for postprandial triglyceride and glycemic responses | Independent external validation (US cohort, r=0.47 for triglyceride, r=0.77 for glucose) |
| 119 | 36,445 tumors across 22 cancer types from three institutions (training); 971 CUP tumors (application) | OncoNPC (machine learning classifier using targeted NGS data) | External validation (held-out tumor samples, weighted F1 score 0.942 for high confidence predictions) |
| 121 | Over 13,000 patients from 16 colorectal cancer cohorts (multicenter) | Transformer-based deep learning pipeline | External validation (trained on surgical resection specimens, validated on endoscopic biopsy tissue; sensitivity 0.99, negative predictive value >0.99 for MSI prediction) |
| 138 | Dataset of 6 initial probiotic-excipient interactions, predicting effects on 111 excipients | Active Machine Learning (with uncertainty sampling) | Experimental validation (correctly predicted 3/4 excipient-probiotic interactions, model certainty 67.70%) |
| 139 | Fecal-inoculated ABIOME system (artificial human GI tract) with probiotic combinations | Multivariate Adaptive Regression Splines (MARS) | Not specified in abstract (algorithm used to identify synergistic probiotic combinations) |
| 145 | Discovery cohort: 130 LO-CRC, 114 EO-CRC, 97 LO-controls, 100 EO-controls; Validation cohort: 38 LO-CRC, 24 EO-CRC, 22 LO-controls, 24 EO-controls | Predictive model based on metagenomic, metabolomic, and KO gene markers | Independent validation cohort |
| 148 | 518 intestinal flora-related genes (text mining) + TCGA database (CRC vs. normal samples) | Protein-protein interaction network analysis (8 key genes identified) | Not specified (bioinformatics analysis with drug-gene interaction database prediction) |
| 159 | 94 colorectal cancer patients (stool samples for 16S rRNA sequencing) | Random Forest (RF) | Not specified in abstract (reported as "qualified efficacy" in predicting KRAS mutation status) |
| 160 | Three published faecal 16S rRNA sequencing datasets | Fuzzy Forest (FF), Random Forest (RF), Recursive Feature Elimination (RFE) | Cross-dataset validation (compared performance across three independent datasets) |
| 166 | More than 700 ICI-treated patient samples across three cancer types (melanoma, gastric cancer, bladder cancer) | NetBio (network-based machine learning framework) | External validation (validated across three independent cancer type cohorts) |
| 174 | Multi-center randomized controlled trial (sample size not specified in title) | Microbiome-based AI-assisted personalized diet model | Randomized controlled trial design |
| 178 | 230 patients undergoing CRC screening or surveillance (8 centers in Italy, UK, US) | Deep learning-based computer-aided detection (CADe) system | Prospective randomized controlled trial design (back-to-back colonoscopies with/without AI) |
| 197 | Discovery cohort: 81 cases (superficial gastritis, atrophic gastritis, intestinal metaplasia, gastric cancer) from Xi'an, China; Validation cohort: 126 cases from Inner Mongolia, China | Five bacterial marker panel (Peptostreptococcus stomatis, Streptococcus anginosus, Parvimonas micra, Slackia exigua, Dialister pneumosintes) | External validation in independent cohort (AUC 0.81 for distinguishing gastric cancer from superficial gastritis) |
| 223 | 192 CRC patients (stages I-II vs. III-IV) | Random Forest (RF) and eXtreme Gradient Boosting (XGBoost) | Not specified in abstract (models effectively distinguished early from late-stage CRC) |
| 231 | 616 participants who underwent colonoscopy | Metagenomic and metabolomic markers | Not specified in abstract (identified markers discriminating intramucosal carcinoma from healthy controls) |
| 232 | 1,368 samples from 8 distinct geographical cohorts | Multi-kingdom (bacterial, fungal, archaeal) diagnostic models | External validation across 3 independent cohorts (AUC = 0.83) |

1. Yamamura K, Baba Y, Nakagawa S, Mima K, Miyake K, Nakamura K, et al. Human Microbiome Fusobacterium Nucleatum in Esophageal Cancer Tissue Is Associated with Prognosis. Clin Cancer Res 2016; 22:5574-81.dio: 10.1158/1078-0432.Ccr-16-1786

2. Feng Q, Liang S, Jia H, Stadlmayr A, Tang L, Lan Z, et al. Gut microbiome development along the colorectal adenoma-carcinoma sequence. Nat Commun 2015; 6:6528.dio: 10.1038/ncomms7528

3. Castaño-Rodríguez N, Goh KL, Fock KM, Mitchell HM, Kaakoush NO. Dysbiosis of the microbiome in gastric carcinogenesis. Sci Rep 2017; 7:15957.dio: 10.1038/s41598-017-16289-2

4. Rugge M, Genta RM, Di Mario F, El-Omar EM, El-Serag HB, Fassan M, et al. Gastric Cancer as Preventable Disease. Clin Gastroenterol Hepatol 2017; 15:1833-43.dio: 10.1016/j.cgh.2017.05.023

5. Parsonnet J, Friedman GD, Vandersteen DP, Chang Y, Vogelman JH, Orentreich N, et al. Helicobacter pylori infection and the risk of gastric carcinoma. N Engl J Med 1991; 325:1127-31.dio: 10.1056/nejm199110173251603

6. Coker OO, Dai Z, Nie Y, Zhao G, Cao L, Nakatsu G, et al. Mucosal microbiome dysbiosis in gastric carcinogenesis. Gut 2018; 67:1024-32.dio: 10.1136/gutjnl-2017-314281

7. Bükki J, Huttner HB, Lee DH, Jantsch J, Janka R, Ostgathe C. Polymicrobial feculent meningitis with detection of Slackia exigua in the cerebrospinal fluid of a patient with advanced rectal carcinoma. J Clin Oncol 2011; 29:e852-4.dio: 10.1200/jco.2011.38.3943

8. Fu K, Cheung AHK, Wong CC, Liu W, Zhou Y, Wang F, et al. Streptococcus anginosus promotes gastric inflammation, atrophy, and tumorigenesis in mice. Cell 2024; 187:882-96.e17.dio: 10.1016/j.cell.2024.01.004

9. Zeller G, Tap J, Voigt AY, Sunagawa S, Kultima JR, Costea PI, et al. Potential of fecal microbiota for early-stage detection of colorectal cancer. Mol Syst Biol 2014; 10:766.dio: 10.15252/msb.20145645

10. Amanati A, Sajedianfard S, Khajeh S, Ghasempour S, Mehrangiz S, Nematolahi S, et al. Bloodstream infections in adult patients with malignancy, epidemiology, microbiology, and risk factors associated with mortality and multi-drug resistance. BMC Infect Dis 2021; 21:636.dio: 10.1186/s12879-021-06243-z

11. Qu R, Zhang Y, Ma Y, Zhou X, Sun L, Jiang C, et al. Role of the Gut Microbiota and Its Metabolites in Tumorigenesis or Development of Colorectal Cancer. Adv Sci (Weinh) 2023; 10:e2205563.dio: 10.1002/advs.202205563

12. de Almeida CV, Taddei A, Amedei A. The controversial role of Enterococcus faecalis in colorectal cancer. Therap Adv Gastroenterol 2018; 11:1756284818783606.dio: 10.1177/1756284818783606

13. Wang N, Fang JY. Fusobacterium nucleatum, a key pathogenic factor and microbial biomarker for colorectal cancer. Trends Microbiol 2023; 31:159-72.dio: 10.1016/j.tim.2022.08.010

14. Alon-Maimon T, Mandelboim O, Bachrach G. Fusobacterium nucleatum and cancer. Periodontol 2000 2022; 89:166-80.dio: 10.1111/prd.12426

15. Huang P, Ji F, Cheung AH, Fu K, Zhou Q, Ding X, et al. Peptostreptococcus stomatis promotes colonic tumorigenesis and receptor tyrosine kinase inhibitor resistance by activating ERBB2-MAPK. Cell Host Microbe 2024; 32:1365-79.e10.dio: 10.1016/j.chom.2024.07.001

16. Rumyantsev KA, Polyakova VV, Sorokina IV, Feoktistova PS, Khatkov IE, Bodunova NA, et al. The Gut Microbiota Impacts Gastrointestinal Cancers through Obesity, Diabetes, and Chronic Inflammation. Life (Basel) 2024; 14.dio: 10.3390/life14101219

17. Wei MY, Shi S, Liang C, Meng QC, Hua J, Zhang YY, et al. The microbiota and microbiome in pancreatic cancer: more influential than expected. Mol Cancer 2019; 18:97.dio: 10.1186/s12943-019-1008-0

18. Şeulean EC, Dumitraşcu DL. The association between exocrine pancreatic insufficiency and changes in gut microbiota: a narrative review. Med Pharm Rep 2025; 98:5-12.dio: 10.15386/mpr-2638

19. Riquelme E, Zhang Y, Zhang L, Montiel M, Zoltan M, Dong W, et al. Tumor Microbiome Diversity and Composition Influence Pancreatic Cancer Outcomes. Cell 2019; 178:795-806.e12.dio: 10.1016/j.cell.2019.07.008

20. Fox JG, Dewhirst FE, Shen Z, Feng Y, Taylor NS, Paster BJ, et al. Hepatic Helicobacter species identified in bile and gallbladder tissue from Chileans with chronic cholecystitis. Gastroenterology 1998; 114:755-63.dio: 10.1016/s0016-5085(98)70589-x

21. Nagaraja V, Eslick GD. Systematic review with meta-analysis: the relationship between chronic Salmonella typhi carrier status and gall-bladder cancer. Aliment Pharmacol Ther 2014; 39:745-50.dio: 10.1111/apt.12655

22. Safaeian M, Gao YT, Sakoda LC, Quraishi SM, Rashid A, Wang BS, et al. Chronic typhoid infection and the risk of biliary tract cancer and stones in Shanghai, China. Infect Agent Cancer 2011; 6:6.dio: 10.1186/1750-9378-6-6

23. Zhou D, Wang JD, Weng MZ, Zhang Y, Wang XF, Gong W, et al. Infections of Helicobacter spp. in the biliary system are associated with biliary tract cancer: a meta-analysis. Eur J Gastroenterol Hepatol 2013; 25:447-54.dio: 10.1097/MEG.0b013e32835c0362

24. Elvevi A, Laffusa A, Gallo C, Invernizzi P, Massironi S. Any Role for Microbiota in Cholangiocarcinoma? A Comprehensive Review. Cells 2023; 12.dio: 10.3390/cells12030370

25. Mathieu A, Leclercq M, Sanabria M, Perin O, Droit A. Machine Learning and Deep Learning Applications in Metagenomic Taxonomy and Functional Annotation. Front Microbiol 2022; 13:811495.dio: 10.3389/fmicb.2022.811495

26. Fiannaca A, La Paglia L, La Rosa M, Lo Bosco G, Renda G, Rizzo R, et al. Deep learning models for bacteria taxonomic classification of metagenomic data. BMC Bioinformatics 2018; 19:198.dio: 10.1186/s12859-018-2182-6

27. Hernández Medina R, Kutuzova S, Nielsen KN, Johansen J, Hansen LH, Nielsen M, et al. Machine learning and deep learning applications in microbiome research. ISME Commun 2022; 2:98.dio: 10.1038/s43705-022-00182-9

28. Li Z, Liu F, Yang W, Peng S, Zhou J. A Survey of Convolutional Neural Networks: Analysis, Applications, and Prospects. IEEE Trans Neural Netw Learn Syst 2022; 33:6999-7019.dio: 10.1109/tnnls.2021.3084827

29. Reiman D, Farhat AM, Dai Y. Predicting Host Phenotype Based on Gut Microbiome Using a Convolutional Neural Network Approach. Methods Mol Biol 2021; 2190:249-66.dio: 10.1007/978-1-0716-0826-5_12

30. Gui X, Bazarova A, Del Amor R, Vieth M, de Hertogh G, Villanacci V, et al. PICaSSO Histologic Remission Index (PHRI) in ulcerative colitis: development of a novel simplified histological score for monitoring mucosal healing and predicting clinical outcomes and its applicability in an artificial intelligence system. Gut 2022; 71:889-98.dio: 10.1136/gutjnl-2021-326376

31. Iacucci M, Cannatelli R, Parigi TL, Nardone OM, Tontini GE, Labarile N, et al. A virtual chromoendoscopy artificial intelligence system to detect endoscopic and histologic activity/remission and predict clinical outcomes in ulcerative colitis. Endoscopy 2023; 55:332-41.dio: 10.1055/a-1960-3645

32. Baranwal M, Clark RL, Thompson J, Sun Z, Hero AO, Venturelli OS. Recurrent neural networks enable design of multifunctional synthetic human gut microbiome dynamics. Elife 2022; 11.dio: 10.7554/eLife.73870

33. Deng X, Li M, Deng S, Wang L. Hybrid gene selection approach using XGBoost and multi-objective genetic algorithm for cancer classification. Med Biol Eng Comput 2022; 60:663-81.dio: 10.1007/s11517-021-02476-x

34. Wei W, Li Y, Huang T. Using Machine Learning Methods to Study Colorectal Cancer Tumor Micro-Environment and Its Biomarkers. Int J Mol Sci 2023; 24.dio: 10.3390/ijms241311133

35. Dai H, Liu Y, Zhu M, Tao S, Hu C, Luo P, et al. Machine learning and experimental validation of novel biomarkers for hypertrophic cardiomyopathy and cancers. J Cell Mol Med 2024; 28:e70034.dio: 10.1111/jcmm.70034

36. Liu J, Huang X, Chen C, Wang Z, Huang Z, Qin M, et al. Identification of colorectal cancer progression-associated intestinal microbiome and predictive signature construction. J Transl Med 2023; 21:373.dio: 10.1186/s12967-023-04119-1

37. Matsuo Y, LeCun Y, Sahani M, Precup D, Silver D, Sugiyama M, et al. Deep learning, reinforcement learning, and world models. Neural Netw 2022; 152:267-75.dio: 10.1016/j.neunet.2022.03.037

38. Botvinick M, Ritter S, Wang JX, Kurth-Nelson Z, Blundell C, Hassabis D. Reinforcement Learning, Fast and Slow. Trends Cogn Sci 2019; 23:408-22.dio: 10.1016/j.tics.2019.02.006

39. Balzerani F, Hinojosa-Nogueira D, Cendoya X, Blasco T, Pérez-Burillo S, Apaolaza I, et al. Prediction of degradation pathways of phenolic compounds in the human gut microbiota through enzyme promiscuity methods. NPJ Syst Biol Appl 2022; 8:24.dio: 10.1038/s41540-022-00234-9

40. Koch M, Duigou T, Faulon JL. Reinforcement Learning for Bioretrosynthesis. ACS Synth Biol 2020; 9:157-68.dio: 10.1021/acssynbio.9b00447

41. Yurdem B, Kuzlu M, Gullu MK, Catak FO, Tabassum M. Federated learning: Overview, strategies, applications, tools and future directions. Heliyon 2024; 10:e38137.dio: 10.1016/j.heliyon.2024.e38137

42. Rozera T, Pasolli E, Segata N, Ianiro G. Machine Learning and Artificial Intelligence in the Multi-Omics Approach to Gut Microbiota. Gastroenterology 2025.dio: 10.1053/j.gastro.2025.02.035

43. Wang N, Cheng M, Ning K. Overcoming regional limitations: transfer learning for cross-regional microbial-based diagnosis of diseases. Gut 2023; 72:2004-6.dio: 10.1136/gutjnl-2022-328216

44. Rong R, Jiang S, Xu L, Xiao G, Xie Y, Liu DJ, et al. MB-GAN: Microbiome Simulation via Generative Adversarial Network. Gigascience 2021; 10.dio: 10.1093/gigascience/giab005

45. Almeida A, Nayfach S, Boland M, Strozzi F, Beracochea M, Shi ZJ, et al. A unified catalog of 204,938 reference genomes from the human gut microbiome. Nat Biotechnol 2021; 39:105-14.dio: 10.1038/s41587-020-0603-3

46. Yachida S, Mizutani S, Shiroma H, Shiba S, Nakajima T, Sakamoto T, et al. Metagenomic and metabolomic analyses reveal distinct stage-specific phenotypes of the gut microbiota in colorectal cancer. Nat Med 2019; 25:968-76.dio: 10.1038/s41591-019-0458-7

47. Liu NN, Jiao N, Tan JC, Wang Z, Wu D, Wang AJ, et al. Multi-kingdom microbiota analyses identify bacterial-fungal interactions and biomarkers of colorectal cancer across cohorts. Nat Microbiol 2022; 7:238-50.dio: 10.1038/s41564-021-01030-7

48. Gou W, Ling CW, He Y, Jiang Z, Fu Y, Xu F, et al. Interpretable Machine Learning Framework Reveals Robust Gut Microbiome Features Associated With Type 2 Diabetes. Diabetes Care 2021; 44:358-66.dio: 10.2337/dc20-1536

49. Roesel R, Strati F, Basso C, Epistolio S, Spina P, Djordjevic J, et al. Combined tumor-associated microbiome and immune gene expression profiling predict response to neoadjuvant chemo-radiotherapy in locally advanced rectal cancer. Oncoimmunology 2025; 14:2465015.dio: 10.1080/2162402x.2025.2465015

50. Yadegar A, Bar-Yoseph H, Monaghan TM, Pakpour S, Severino A, Kuijper EJ, et al. Fecal microbiota transplantation: current challenges and future landscapes. Clin Microbiol Rev 2024; 37:e0006022.dio: 10.1128/cmr.00060-22

51. Ooijevaar RE, Terveer EM, Verspaget HW, Kuijper EJ, Keller JJ. Clinical Application and Potential of Fecal Microbiota Transplantation. Annu Rev Med 2019; 70:335-51.dio: 10.1146/annurev-med-111717-122956

52. Cunningham M, Azcarate-Peril MA, Barnard A, Benoit V, Grimaldi R, Guyonnet D, et al. Shaping the Future of Probiotics and Prebiotics. Trends Microbiol 2021; 29:667-85.dio: 10.1016/j.tim.2021.01.003

53. Di Luccia B, Colonna M. Precision Probiotic Medicine to Improve ICB Immunotherapy. Cancer Discov 2022; 12:1189-90.dio: 10.1158/2159-8290.Cd-22-0221

54. Ul Ain N, Naveed M, Aziz T, Shabbir MA, Al Asmari F, Abdi G, et al. Mix-match synthesis of nanosynbiotics from probiotics and prebiotics to counter gut dysbiosis via AI integrated formulation profiling. Sci Rep 2024; 14:18397.dio: 10.1038/s41598-024-69515-z

55. Wang T, Yin Q, Huang HY, Wang Z, Song H, Luo X. Probiotic Escherichia coli Nissle 1917 propelled micro-robot with pH sensitivity for hypoxia targeted intestinal tumor therapy. Colloids Surf B Biointerfaces 2023; 225:113277.dio: 10.1016/j.colsurfb.2023.113277

56. Zhao M, Jiang Y, Chen Z, Fan Z, Jiang Y. Traditional Chinese medicine for Helicobacter pylori infection: A protocol for a systematic review and meta-analysis. Medicine (Baltimore) 2021; 100:e24282.dio: 10.1097/md.0000000000024282

57. Liu BG, Xie M, Dong Y, Wu H, He DD, Hu GZ, et al. Antimicrobial mechanisms of traditional Chinese medicine and reversal of drug resistance: a narrative review. Eur Rev Med Pharmacol Sci 2022; 26:5553-61.dio: 10.26355/eurrev_202208_29426

58. Ross FC, Patangia D, Grimaud G, Lavelle A, Dempsey EM, Ross RP, et al. The interplay between diet and the gut microbiome: implications for health and disease. Nat Rev Microbiol 2024; 22:671-86.dio: 10.1038/s41579-024-01068-4

59. Wastyk HC, Fragiadakis GK, Perelman D, Dahan D, Merrill BD, Yu FB, et al. Gut-microbiota-targeted diets modulate human immune status. Cell 2021; 184:4137-53.e14.dio: 10.1016/j.cell.2021.06.019

60. Zmora N, Suez J, Elinav E. You are what you eat: diet, health and the gut microbiota. Nat Rev Gastroenterol Hepatol 2019; 16:35-56.dio: 10.1038/s41575-018-0061-2

61. Mann ER, Lam YK, Uhlig HH. Short-chain fatty acids: linking diet, the microbiome and immunity. Nat Rev Immunol 2024; 24:577-95.dio: 10.1038/s41577-024-01014-8

62. Divya Ganeshan S, Hosseinidoust Z. Phage Therapy with a Focus on the Human Microbiota. Antibiotics (Basel) 2019; 8.dio: 10.3390/antibiotics8030131

63. Neil K, Allard N, Roy P, Grenier F, Menendez A, Burrus V, et al. High-efficiency delivery of CRISPR-Cas9 by engineered probiotics enables precise microbiome editing. Mol Syst Biol 2021; 17:e10335.dio: 10.15252/msb.202110335
